# Shared memories of event details in the human brain are altered by misinformation and test expectations

**DOI:** 10.1371/journal.pbio.3003886

**Published:** 2026-07-06

**Authors:** Xuhao Shao, Chuansheng Chen, Elizabeth F. Loftus, Bi Zhu

**Affiliations:** 1 State Key Laboratory of Cognitive Neuroscience and Learning, Beijing Normal University, Beijing, China; 2 Institute of Developmental Psychology, Beijing Normal University, Beijing, China; 3 IDG/McGovern Institute for Brain Research, Beijing Normal University, Beijing, China; 4 Department of Psychology, University of California, Irvine, California, United States of America; Radboud Universiteit Donders Institute for Brain Cognition and Behaviour, NETHERLANDS, KINGDOM OF THE

## Abstract

Shared memories of event details are crucial to eyewitness testimony. When different people encode or recall the same event, similar scene-specific neural activity patterns emerge across individual brains. However, it remains unclear whether these patterns are specific to event details and how test expectancy (i.e., expecting free recall or general memory tests) and misinformation affect them. In this study, 100 participants were randomly assigned to view one of two versions of each event. Both versions featured identical scenarios, but with different details. About half of the participants were informed about the upcoming free recall before viewing events, while the others were told to expect a general memory test. Functional magnetic resonance imaging was used to record their brain activity during four stages: viewing original events, initial free recall, reading misinformation, and final free recall of original events. The neuroimaging data were analyzed based on the similarity of neural patterns across participants. Test expectancy increased the similarity of detail-specific neural activity patterns between individuals when they viewed original events in brain regions relevant for visual attention. Misinformation increased the likelihood of people forming shared false memories of event details. People who formed shared false memories exhibited similar detail-specific patterns of activity in the dorsomedial prefrontal cortex when reading misinformation. People who formed shared true memories exhibited similar detail-specific patterns of activity in the inferior parietal lobe when viewing original events, as well as in the ventrolateral prefrontal cortex and middle temporal gyrus when recalling them after exposure to misinformation. Our findings revealed that different brain regions of the default mode network play distinct roles in the encoding and recall of event details shared by individuals.

## Abstract 2

Shared neural patterns across individuals support memory for event details, but how test expectancy and misinformation shape these representations remains unclear. This study shows that expectancy enhances encoding similarity in brain regions relevant for visual attention, while true and false memories activated different brain regions of the default mode network, showing they play distinct roles in the encoding and recall of event details shared by individuals.

## Introduction

Memories of events are typically composed of multiple scenes, each containing various details such as people and locations. We may form shared memories of specific scenes within an event, as well as shared memories of particular details within those scenes [1–5]. For example, multiple witnesses may see a criminal hiding and later recall this scene. However, only some will encode the precise location of the hiding place, and a few will ultimately recall this detail. When multiple witnesses recall identical details, their testimonies are more likely to be considered credible. Therefore, it is of great importance to understand how such shared memories are instantiated across individuals. Previous research has shown that, when different individuals encode or recall the same event scenes, similar patterns of neural activity emerge between their brains, particularly in their posterior medial cortex [2,6–8]. However, it is unclear whether these patterns are specific to event details. Furthermore, the impact of pre-event test expectations (e.g., expecting free recall or general memory tests) and post-event misinformation on them is not well understood.

Pre-event expectations of free recall tests may enhance the encoding of specific event details shared between individuals. According to the encoding strategy adaptation hypothesis [9–11], individuals’ expectations about testing can alter their encoding strategies, thereby affecting subsequent memory performance. Specifically, if individuals know in advance that they will undergo a free recall test after viewing an event, they are more likely to focus their visual attention on the details of the event during the encoding process and to integrate the information into a narrative form. Behavioral studies have shown that knowing the type of test in advance improves the encoding and retrieval of event details [12–14]. In the previous neuroimaging studies, however, researchers provided participants with various types of task instructions before encoding events [15–17]. Some studies informed participants about the free recall test prior to the encoding of events [2,6,18,19]. Other studies informed participants that they would be tested on the content of events after encoding, though they did not specify that it would be a recall test [8,20]. Still other studies asked participants to encode events without requiring them to take a test afterward [21–23]. However, none of these studies examined how pre-event expectations of free recall tests impacted the way event details are encoded and shared across individual brains.

Post-event misinformation provided by other witnesses may also alter the recall of specific event details shared between individuals. About a century ago, Bartlett [24] discovered that humans spontaneously generate false memories when repeatedly recalling stories. False memories can be further increased by post-event misinformation [25–27]. Moreover, misinformation can lead to false memories of specific details that are shared among people [27–30]. Investigating how misinformation alters shared memories of event details has implications for understanding the reliability of eyewitness testimony and the societal impact of fake news [31–35]. If multiple witnesses all report the same misinformation in their free recalls, that misinformation is more likely to be mistaken for the truth [36–38]. This phenomenon of shared false memories is known as “the Mandela effect”, named after the collective false memory shared by many that Nelson Mandela died in prison in the 1980s [29]. Recent behavioral studies have confirmed that shared false memories emerge spontaneously during recall of visual icons [39,40]. Prior research has also revealed that individuals’ recognition of event details can be influenced by the misinformation provided by co-witnesses [41,42]. However, no research has yet explored how individuals develop shared false memories of specific event details during free recall after exposure to misinformation.

Previous studies have examined the neural basis of individual false recognition induced by misinformation [41,43–47]. Some studies employed the classic three-stage misinformation paradigm: viewing original events, exposure to misinformation, and recognition tests of original events [45–47]. Other studies added an initial test to measure recognition performance before exposure to misinformation [41,43,44]. Researchers have compared differences in brain activity levels during encoding or retrieval between true and false recognition [41,45,47], and between participants who received and those who did not receive a warning about misinformation [43,44]. In our previous studies, we used representational similarity analysis to calculate the intra-subject similarity of neural activity patterns between encoding and recognition at the scene-specific level. We found that the hippocampus and posterior medial cortex carried participant-specific representations that predicted false recognition induced by misinformation [46]. However, shared free recall differs from individual recognition in a number of ways. In contrast to recognition, free recall of event details relies more on recollection and verbal organizational processes and less on external retrieval cues [1,48–50]. People can recognize alternative answers based on familiarity, but when freely recalling events, they must exert more effort to retrieve memory traces of details and reconstruct them verbally. Thus, the neural mechanisms underlying shared false recall and individual false recognition of event details may differ.

**Fig 1:**
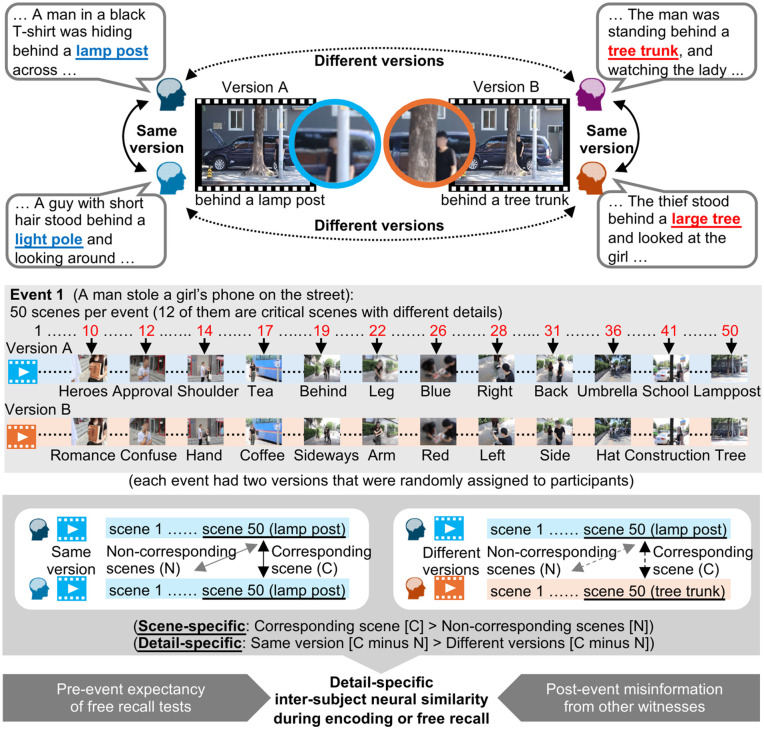
Detail-specific representations shared by individuals during event encoding and free recall. When different people encode and recall the same event, they are most likely to collectively remember specific scenes (e.g., a man hiding). However, a few people may also collectively remember specific details (e.g., hiding behind a big tree or tree trunk, depending on individuals’ labels of the detail in their recall). To investigate the detail-specific representations that are shared by individuals, we created two versions of the materials for each event. Each event consists of 50 scenes (images), including 12 critical scenes that have two versions each. Both versions featured the same plot but with different details. Participants were randomly assigned to watch one of the two versions of each event. The images in this figure have been blurred to protect personal privacy, but the images viewed by participants were clear. Scene-specific neural representations shared by individuals were indicated by greater inter-subject neural pattern similarity for the corresponding scene (C) than the average similarity value for the remaining non-corresponding scenes in the same event (N) (i.e., C > N). Based on this, detail-specific representations shared by individuals were indicated by greater inter-subject scene-specific neural pattern similarity for the same version than that for different versions (i.e., same version [C minus N] > different versions [C minus N]). We explored which brain regions carried detail-specific representations that were shared by individuals during encoding or free recall. We also examined how test expectancy and post-event misinformation influenced these representations.

The present study investigated which brain regions carry the detail-specific representations that are shared by individuals during encoding or free recall. To investigate detail-specific representations, we created two versions of materials for each event featuring the same plot but with different details. For example, about half of the participants saw a man hiding behind a lamp post, while the other participants saw a man hiding behind a tree trunk (Fig 1). If participants who observed the same details exhibited greater neural similarity than participants who observed different details in a corresponding scene of an event, then detail-specific representations were indicated. We then examined how these detail-specific representations were influenced by test expectancy and post-event misinformation.

To investigate how test expectancy of free recall would influence the encoding of event details that were shared across individuals, 100 participants were divided into two groups and given different instructions before viewing events. Specifically, the participants in the recall group were informed, before viewing events, that they would take free recall tests. The participants in the control group were told that they would take memory tests, but the tests were not specified as free recall. The participants in the control group of this study were previously reported in our earlier study [46], where these data were used for within-subject analysis. In this study, we used the inter-subject neural similarity analysis to examine neural representations shared across participants within each group. Then, we examined the test expectancy effect by comparing the inter-subject similarity data between the recall group and the control group. According to the encoding strategy adaptation hypothesis, the expectation of free recall tests may promote perceptual, semantic, and associative processing of event details. Thus, participants in the recall group would exhibit greater similarity in detail-specific neural activity patterns in brain regions involved in visual attention and default mode networks when viewing original events than participants in the control group. As predicted, expectations of free recall tests increased the similarity of detail-specific neural activity patterns between individuals when they viewed original events.

To investigate how post-event misinformation would influence the recall of event details that were shared across individuals, participants in the recall group viewed the original events, performed the initial free recall of the events, read post-event narratives containing misinformation, and performed the final free recall of the original events. We examined whether people who formed shared false memories exhibited similar detail-specific patterns of activity when reading misinformation, and whether people who formed shared true memories exhibited similar detail-specific patterns of activity when viewing original events. Finally, we investigated whether people who formed shared true or false memories exhibited similar detail-specific patterns of neural activity during the final free recall after exposure to misinformation. As expected, misinformation increased the likelihood that people would form shared false memories of event details. They were supported by detail-specific neural representations that were shared across individuals. Our findings elucidate how test expectations and post-event misinformation alter shared neural representations among individuals. Given the reconstructive nature of human memory, the spreading of misinformation online, and the prevalence of large-scale interactions on social media, research in this area holds both theoretical and practical significance [51–53].

## Results

### Test expectation enhanced the encoding of event details shared across brains

**Fig 2:**
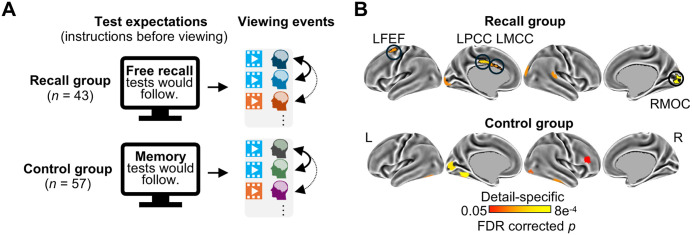
The expectation of free recall tests enhanced the encoding of event details shared across individual brains. **(A)** Manipulation of participants’ expectations of tests (between-subjects design: Recall vs. Control). Before the original events were presented, the participants in the recall group were informed that they would take free recall tests. In contrast, the participants in the control group were only told that they would take memory tests without being informed of the specific type. Then, both groups of participants viewed all eight original events while undergoing fMRI scanning. Approximately half of the participants in each group saw version A of the events, and the other half saw version B. We examined the detail-specific inter-subject neural pattern similarity between participants within each group during the encoding of original events. Then, we investigated whether the recall group showed greater inter-subject neural pattern similarity than did the control group in response to specific event details during the encoding of original events. **(B)** Detail-specific neural pattern similarity between participants within each group during the encoding of original events (outlined ROIs: Recall > Control). In the recall group, detail-specific representations shared by all participants were found in the visual, auditory, temporal, frontal, and cingulate cortices. In the control group, detail-specific representations shared by all participants were primarily located in the visual and frontal cortices. Compared to the control group, the recall group exhibited stronger detail-specific representations in the left frontal eye fields (LFEF), left posterior cingulate cortex (LPCC), left middle cingulate cortex (LMCC), and right medial occipital cortex (RMOC). No brain region showed stronger detail-specific representations in the control group than in the recall group. L: left. R: right. The underlying numerical data for this figure are provided in S1 Data.

First, we analyzed the fMRI data of the recall and control groups while they encoded original events (Fig 2A, see the Methods section for details). In each group, the detail-specific representation was indicated by greater inter-subject scene-specific neural pattern similarity for the same version than for different versions. These analyses were conducted for all critical scenes encoded by each group’s participants, regardless of their subsequent behavioral performance. In the recall group (Fig 2B [top] and S1 Table), detail-specific representations shared by all participants were found in the visual, auditory, temporal, frontal, and cingulate cortices (FDR-corrected *p* < 0.05). In the control group (Fig 2B [bottom] and S2 Table), detail-specific representations shared by all participants were primarily located in the visual and frontal cortices (FDR-corrected *p* < 0.05). These results remained significant in permutation analyses based on 10,000 random shuffles (permuted FDR-corrected *p* < 0.05, as shown in S1 and S2 Tables).

Then, we examined the test expectancy effect as indicated by greater inter-subject detail-specific neural pattern similarity in the recall group than in the control group. The test expectancy effect was found in the following brain regions: the LFEF, the LPCC, the LMCC, and the RMOC (*t*(98) = 4.52, 4.65, 3.92, and 3.86, FDR-corrected *p* = 0.003, 0.003, 0.017, and 0.017; shown as outlined ROIs in Fig 2B [top] and see S3 Table for details). These results remained significant in permutation analyses (permuted FDR-corrected *p* < 0.05, as shown in S3 Table). The mean and standard deviation of the inter-subject similarity value for each of these regions are presented in S4 Table. No brain region showed stronger detail-specific representations in the control group than in the recall group. The raw *p*-values and FDR-corrected *p*-values for the 400 regions covering the entire cortex are provided in the S1 Data.

### Misinformation-induced shared false memories of event details during free recall

#### Critical scenes recalled before or after exposure to misinformation.

**Fig 3:**
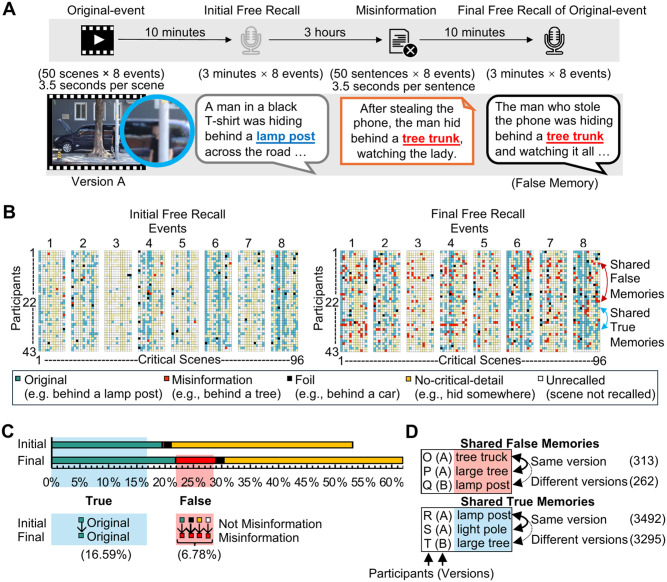
Misinformation-induced shared false memories of event details during free recall. **(A)** Experimental procedure for the recall group. Participants completed a four-stage task inside the fMRI scanner. They first viewed original events, followed by an initial free recall test. Later, they were informed they would read other witnesses’ narratives about these events. However, they were not told that these narratives contained misinformation that was inconsistent with the original events. After reading the misinformation, some participants recalled misinformation in the final test (i.e., false memory), despite being instructed to recall based on what they had seen in the original events. Each participant’s spoken recall in each free recall test was transcribed, segmented, and labeled as one of the 50 scenes for each event. **(B)** Critical scenes recalled before and after exposure to misinformation. Each square represents the recall performance for each of the combination of 12 critical scenes by 8 events by 43 participants. There are four types of recall, including original (i.e., recalling the scene with critical details from the original events), misinformation (i.e., recalling the scene with critical details from misinformation), foil (i.e., recalling the scene with critical details but from neither the original events nor misinformation), and no-critical-detail (i.e., recalling the scene without critical details). If the scene was not recalled at all, it was coded as unrecalled. The misinformation effect was evident as participants reported more misinformation than foils in the final recall. And their final recall contained more misinformation than the initial recall. **(C)** Persistent true memory and misinformation-induced false memory. Persistent true memory (True): recalling the original detail for the corresponding scene in both the initial and final recall. Misinformation-induced false memory (False): recalling misinformation in the final recall but not in the initial recall for the corresponding scene. **(D)** Shared memories in the final recall after exposure to misinformation (the total number of inter-subject pairs). Shared false memories occurred when pairs of participants had misinformation-induced false memories about specific event details in the final recall (e.g., participants O, P, and Q). Regarding shared false memories, the total number of inter-subject pairs of participants who viewed the same version (e.g., participants O and P) and different versions (e.g., participants O and Q) was 313 and 262, respectively. Shared true memories occurred when pairs of participants had persistent true memory about specific event details in the final recall (e.g., participants R, S, and T). Regarding shared true memories, the total number of inter-subject pairs of participants who viewed the same version (e.g., participants R and S) and different versions (e.g., participants R and T) was 3,492 and 3,295, respectively. The underlying numerical data for this figure are provided in S1 Data.

After viewing the original events, the participants in the recall group engaged in an initial free recall. Then, they read misinformation that was inconsistent with the original events. Finally, they engaged in a final free recall of the original events. Participants’ initial and final free recalls of the critical scenes were coded into four types: original (i.e., recalling the scene with critical details from the original events), misinformation (i.e., recalling the scene with critical details from misinformation), foil (i.e., recalling the scene with critical details but from neither the original events nor misinformation), and no-critical-detail (i.e., recalling the scene without critical details) (Fig 3A and S5 Table). If the scene was not recalled at all, it was coded as unrecalled. A 2 (recall stage: initial versus final) by 4 (recall type: original, misinformation, foil, and no-critical-detail) repeated measures ANOVA revealed misinformation effects in free recall. There was a significant interaction between recall stage and recall type (*F*(3, 126) = 17.58, *p* = 1e^−9^, *η_p_*^2^ = 0.30) (S1 Fig and S6 Table). This effect was mainly due to the greater increase in misinformation recall from the initial to the final tests compared to the other three types of recall (*ps* < 9e^−4^). The most direct evidence for the misinformation effect was that misinformation recall was lower than foil recall in the initial test, but higher in the final test (*ps* < 8e^−4^).

#### Persistent true memory and misinformation-induced false memory.

Since the initial and final recalls contained overlapping and distinct details for each recall type, the performance of the final recall must be refined based on whether specific details persisted between the two recalls (Fig 3B and S7 Table). Persistent true memory was defined as recalling the corresponding scene with critical details of the original events in both the initial and final tests. Misinformation-induced false memory was defined as recalling the corresponding scene with critical details of the misinformation in the final test but not in the initial test. In the final test, the percentages of persistent true memory and misinformation-induced false memory were 16.59% and 6.78%, respectively.

#### Shared true memories and shared false memories after exposure to misinformation.

Shared misinformation-induced false memories (shortened as shared false memories hereafter) were defined as situations when a pair of participants recalled the misinformation of specific event details only in the final test (Fig 3C). Shared false memories were observed in a total of 313 inter-subject pairs of participants who had viewed the same version of the critical scenes, as compared to 262 inter-subject pairs of participants who had viewed different versions of the critical scenes. Shared true memories were defined as situations when a pair of participants recalled the specific original details of the corresponding scene in both the initial and final tests. Shared true memories were observed in a total of 3,492 inter-subject pairs of participants who had viewed the same version of the critical scenes, as compared to 3,295 inter-subject pairs of participants who had viewed different versions of the critical scenes. Subsequent analyses of shared true or false memories were conducted by computing inter-subject similarities for the same and different versions separately at the trial level (i.e., for each pair of participants). Values from different versions were used as baselines for comparison with values from the same version. Additional behavioral results are shown in S8 and S9 Tables. All underlying behavioral data for these analyses are provided in the S1 Data.

### Shared neural representations of original details for shared true memories

**Fig 4:**
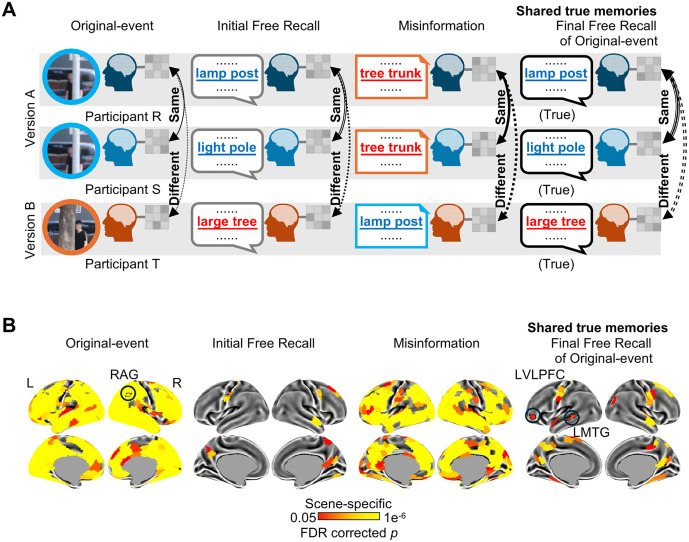
Shared neural representations for shared true memories. **(A)** The inter-subject similarity for shared true memories. Scene-specific representation was indicated by greater inter-subject similarity for the corresponding scene than the average similarity value for the noncorresponding scenes in the same event. Detail-specific representation was indicated by greater inter-subject scene-specific similarity for the same version (solid curves) than that for different versions (dashed curves). **(B)** Scene-specific representations were found in multiple brain regions (e.g., the posterior medial cortex) during each of the four stages for participants with shared true memories after viewing the same version (color-coded). Far left: Right angular gyrus (RAG) exhibited detail-specific representations during the encoding of original events (outlined ROI). Center-left: No cortical region exhibited detail-specific representations during the initial free recall. Center-right: No cortical region exhibited detail-specific representations during the encoding of misinformation. Far right: Left ventrolateral prefrontal cortex (LVLPFC) and left middle temporal gyrus (LMTG) exhibited detail-specific representations during the final free recall (outlined ROIs). These results suggest that people who form shared true memories have similar detail-specific patterns of activity in the RAG when encoding original events. They also have similar detail-specific patterns of activity in the left ventrolateral prefrontal cortex and middle temporal gyrus when recalling the events after exposure to misinformation. L: left. R: right. The underlying numerical data for this figure are provided in S1 Data.

We investigated whether participants with shared true memories after exposure to misinformation possessed shared neural representations for specific scenes and details within those scenes (Fig 4A). First, we explored which brain regions of the participants who shared true memories after viewing the same version exhibited scene-specific representations during each stage. Then, we tested whether these regions exhibited detail-specific representations during each stage. To do that, we computed the scene-specific and detail-specific similarity of neural activity patterns between participants with shared true memories (see the Methods section for details).

During the encoding of original events (Fig 4B [far left]), the majority of cortical regions had similar scene-specific patterns of activity between participants with shared true memories after viewing the same version (FDR-corrected *p* < 0.05). However, only the right angular gyrus (RAG) exhibited similar detail-specific patterns of activity between participants with shared true memories (*t*(6073.81) = 4.27, FDR-corrected *p* = 0.008). This result remained significant in permutation analyses (permuted FDR-corrected *p* = 0.040). This suggests that when individuals encode original-event details, the RAG exhibits shared representations across individuals, resulting in the formation of shared true memories.

During the initial free recall (Fig 4B [center-left]), the posterior medial cortex and a few frontotemporal regions had similar scene-specific patterns of activity between participants with shared true memories after viewing the same version (FDR-corrected *p* < 0.05), but none exhibited similar detail-specific patterns. During the encoding of misinformation (Fig 4B [center-right]), the majority of cortical regions had similar scene-specific patterns of activity between these participants (FDR-corrected *p* < 0.05), but none of these patterns were detail-specific.

During the final free recall, the posterior medial cortex and several frontotemporal regions still had similar scene-specific patterns of activity between participants with shared true memories after viewing the same version (FDR-corrected *p* < 0.05). Detail-specific representations shared by participants with shared true memories were observed in the left ventrolateral prefrontal cortex (*t*(5782.04) = 3.70, FDR-corrected *p* = 0.044) and the left middle temporal gyrus (LMTG) (*t*(5857.41) = 3.71, FDR-corrected *p* = 0.044) (Fig 4B [far right]). These results remained significant in permutation analyses (permuted FDR-corrected *ps* = 0.040). This suggests that when individuals persistently recall original details after exposure to misinformation, shared representations across individuals in these regions are associated with shared true memories. The mean and standard deviation of the inter-subject similarity value for each of these regions are presented in S10 Table. The raw *p*-values and FDR-corrected *p*-values for the 400 regions covering the entire cortex are provided in the S1 Data.

### Shared neural representations of misinformation for shared false memories

**Fig 5:**
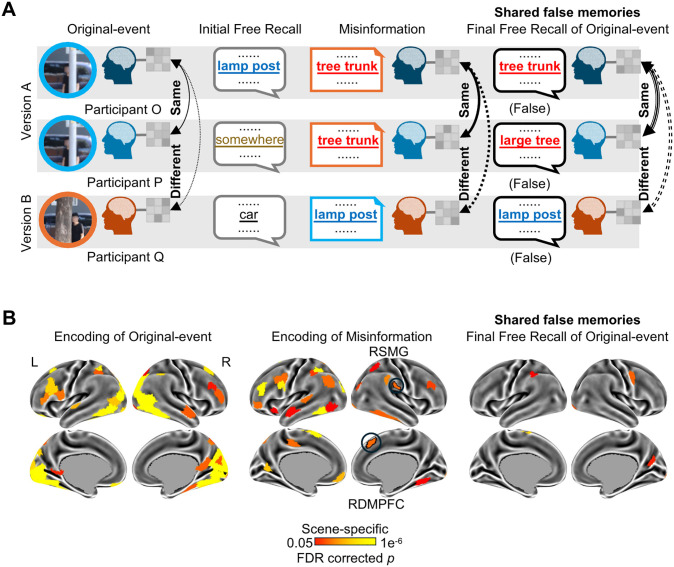
Shared neural representations for shared false memories. **(A)** Inter-subject similarity for shared false memories. Scene-specific representation was indicated by greater inter-subject similarity for the corresponding scene than the average similarity value for the noncorresponding scenes in the same event. Detail-specific representation was indicated by greater inter-subject scene-specific similarity for the same version (solid curves) than that for different versions (dashed curves). **(B)** Scene-specific representations were found in multiple brain regions during each of the three stages for participants with shared false memories after viewing the same version (color-coded). During the encoding of misinformation, detail-specific representations were observed in the right dorsomedial prefrontal cortex (RDMPFC) and the right supramarginal gyrus (RSMG) (outlined ROIs). This suggests that when individuals encode misinformation, these brain regions exhibit shared representations across individuals, resulting in the formation of shared false memories. However, no cortical regions exhibit detail-specific representations during the encoding and final recall of original events. The underlying numerical data for this figure are provided in S1 Data.

Next, we examined shared false memories using a similar analytical approach (Fig 5A). We examined inter-subject similarity between participants with shared false memories for each of the following three stages: encoding of the original events, encoding of the misinformation, and the final recall test (see the Methods section for details). The initial free recall stage was not examined here because, unlike shared true memories, shared false memories only appeared in the final recall after misinformation had been introduced.

During the encoding of original events (Fig 5B [left]), the frontoparietal and visual cortex had similar scene-specific patterns of activity between participants with shared false memories after viewing the same version (FDR-corrected *p* < 0.05), but none showed detail-specific patterns. During the encoding of misinformation (Fig 5B [middle]), the right dorsomedial prefrontal cortex (*t*(564.63) = 3.92, FDR-corrected *p* = 0.020) and the right supramarginal gyrus (*t*(571.98) = 4.14, FDR-corrected *p* = 0.016) had similar detail-specific patterns of activity between participants with shared false memories. In permutation analyses, the result regarding the right dorsomedial prefrontal cortex remained significant (permuted FDR-corrected *p* = 0.040), whereas the result regarding the right supramarginal gyrus reached marginal significance (permuted FDR-corrected *p* = 0.059). This suggests that when individuals encode misinformation, these brain regions exhibit shared representations across individuals, resulting in the formation of shared false memories. During the final free recall, no brain region showed detail-specific patterns (Fig 5B [right]). The mean and standard deviation of the inter-subject similarity value for each of these brain regions are presented in S11 Table. The raw *p*-values and FDR-corrected *p*-values for the 400 regions covering the entire cortex are provided in the S1 Data. Additional results related to the hippocampus are presented in S12 Table.

## Discussion

This study found that participants exhibited similar (i.e., shared) patterns of neural activity when encoding or recalling specific event details, and these patterns were influenced by test expectancy and post-event misinformation. When encoding original events, brain regions involved in visual attention exhibited similar detail-specific activity patterns across all individuals. This shared encoding was further enhanced when participants were informed in advance that free recall tests would follow the event encoding. For participants who formed shared true or false memories after exposure to misinformation, distinct brain regions associated with the default mode network exhibited inter-subject similarity during the encoding or recall of original details or misinformation. Specifically, for individuals with shared true memories, detail-specific representations emerged in the inferior parietal lobe during encoding of original events, and were then observed in the ventrolateral prefrontal cortex and middle temporal gyrus during recall of original events after exposure to misinformation. For individuals with shared false memories, detail-specific representations emerged in the dorsomedial prefrontal cortex during encoding of misinformation. This suggests that misinformation can alter shared memories of event details that originate from shared neural representations during the encoding of either the original events or the misinformation.

Building upon the scene-specific inter-subject neural similarity observed in previous studies [2,6–8], we present new evidence suggesting the existence of detail-specific inter-subject neural similarity. Notably, all participants exhibited similar detail-specific neural activity patterns in their visual cortex when encoding events, regardless of whether they were asked to recall or were able to recall specific details. Interestingly, this shared encoding of details was more pronounced when participants were informed in advance that they would undergo free recall tests. In support of the encoding strategy adaptation hypothesis [9–11], our findings suggest that test expectancy enhances the shared encoding of event details in the visual cortex and the medial and lateral frontoparietal regions. Most of these brain regions are located in the visual, dorsal attention, and default mode networks [54–56]. The anticipation of upcoming free recall tests seems to synchronize how individuals attend to specific details within an event scene. For participants in the recall group, the expectation of free recall tests likely encouraged elaborative encoding of event details, resulting in similar activity patterns among individuals. In contrast, participants in the control group were likely to employ more flexible memory encoding strategies, resulting in idiosyncratic activity patterns. This is because they could not preemptively tailor their encoding strategies to accommodate particular test formats, such as recognition, cued recall, or free recall. We should point out that since most witnesses are unaware of future recall tests when experiencing real-life events, their neural activity patterns during event encoding may be less similar than those observed in experimental settings where participants are informed of recall tests beforehand.

Our behavioral findings from repeated free recall tests not only confirm the impact of misinformation on individual memory [30,57,58], but also reveal how misinformation induces people to form shared false memories about specific event details. Specifically, recall of misinformation increased more than recall of any other type when comparing the free recall of original events before and after exposure to misinformation. It is worth noting that despite the significant amount of misinformation-induced false memory (~7% of critical details), the amount of persistent true memories was still greater (i.e., participants accurately and consistently recalled around 17% of critical details in the final test). In terms of shared memories, participants demonstrated both shared false memories induced by misinformation and shared true memories that persisted even after exposure to misinformation. Our results on shared false memory provide empirical evidence for the Mandela effect [29,39], namely, that individuals develop shared false memories after exposure to misinformation.

During encoding, detail-specific representations in the right inferior parietal cortex were found to predict subsequent shared memories of these details. When encoding original events, individuals with shared true memories exhibited similar detail-specific activity patterns in their right angular gyrus. When encoding post-event misinformation, individuals with shared false memories exhibited similar detail-specific activity patterns in their right supramarginal gyrus. This indicates that precise event details encoded by different people are represented by shared neural signatures in the inferior parietal cortex. Such cross-person convergence suggests that the inferior parietal lobe functions as a mnemonic hub that integrates perceptual and conceptual information into a coherent representation of event details. This interpretation aligns with extensive evidence that the lateral parietal cortex represents vivid episodic content and event features [59–62]. Our results extend these findings by showing that shared detail-specific encoding within the right inferior parietal lobe predicts later shared recall of those details. This result emphasizes this region’s role in creating shared neural representations that encode event details across individuals.

Expanding on prior research concerning the neural basis of individual false recognition induced by misinformation [41,43–47], our study suggests that misinformation is encoded in the dorsomedial prefrontal cortex in a way that is shared across individuals, leading to shared false recall. The dorsomedial prefrontal cortex appears to play a key role in the encoding of misinformation provided by other people. In our study, participants were told that they would read verbal descriptions of these events provided by other witnesses. When people learn from others’ memories of these events, they may adopt an evaluative stance to avoid acquiring misinformation [63,64]. However, if misinformation is mistakenly judged as credible, it can result in the formation of false memories. Previous studies have shown that the dorsomedial prefrontal cortex supports an individual’s ability to understand others’ thoughts during social interactions [65–67]. Our results build on these findings by demonstrating that individuals with shared false memories exhibit similar activity patterns when misinformation is encoded in the dorsomedial prefrontal cortex.

During free recall, the posterior medial cortex appears to play a key role in the shared recall of event scenes, while the left frontotemporal cortex seems to be crucial for the accurate shared recall of event details. In line with prior research examining neural similarity among individuals [2,6–8], participants demonstrated similar scene-specific activity patterns in the posterior medial cortex during free recall. However, only the activity patterns in the left ventrolateral prefrontal cortex and the left middle temporal gyrus exhibited detail-specific inter-subject similarity among individuals with shared true memories during the final free recall after exposure to misinformation. Previous studies suggest that these two cortical regions are selectively involved in the recall of conceptual details [68–70]. Interestingly, detailed representations were only observed during the final recall after exposure to misinformation. This suggests that the introduction of misinformation can actually improve true memory of certain details from the original events [71,72]. These findings indicate that shared true memories after exposure to misinformation rely on an evolved neural network that enables different individuals to encode and then recall the same event details with remarkable consistency.

Future research should address several limitations of this study. First, we examined the neural basis of shared false memories induced by misinformation in individuals engaged in free recall alone in an fMRI scanner. Future hyperscanning studies could further examine how people’s brains synchronize when sharing misinformation face-to-face. Second, we used a design of repeated recall separated by a few hours. However, if the interval is longer than one day, the dis-allocation of engram neurons may allow these two memories to be stored separately [72,73]. According to the schema and fuzzy-trace theories of memory [24,74], true recall of event details tends to decline, whereas spontaneous false memories increase with longer intervals between repeated tests. Future research could investigate the neural basis of long-lasting false memories shared by individuals. Third, there has been controversy as to whether recalling details of an event immediately after it occurs increases later susceptibility to misinformation [75,76]. Furthermore, behavioral researchers have found that the initial test allocates more attention resources to the encoding of post-event misinformation [77]. Therefore, the neural findings we obtained during the misinformation stage may have been influenced by the initial recall. Information that people recalled during the initial test may be more likely to be recalled again during the final test. Conversely, information that individuals did not recall during the initial test may be more likely to be forgotten during the final test. Future research could build on this study by including an additional recall group without initial tests, thereby ruling out the potential influence of initial recall on neural representations during the misinformation stage. Then, the two groups could be directly compared to explore the neural basis of retrieval-enhanced suggestibility. Additionally, our findings may be related to the type of misinformation used in the study. Our study included five types of misleading details, most of which were related to objects and text. Future research could focus on specific types of details to investigate how different types of details influence shared false memories and their neural representations. Such work may also benefit from larger sample sizes, which could improve sensitivity to detect additional brain regions involved in the neural representation of shared false memories. Finally, this study employed time-limited recall tasks to ensure standardization. Most participants were able to recall each event within the given time. However, a small number of them might have felt rushed and were unable to share all the details they knew. A small number of them might have stopped speaking before the time limit ended, even though they might still be recalling the event in their minds. Future studies could use self-paced recall tasks.

## Methods

### Ethics statement

This study was approved by the Institutional Review Board at State Key Laboratory of Cognitive Neuroscience and Learning at Beijing Normal University, China (approval no. IRB_A_0039_2023001) and conducted in accordance with the Declaration of Helsinki. Participants provided written informed consent and were paid 100 Chinese yuan (about USD $15) per hour.

### Participants

Participants in the recall and control groups were recruited by the same criteria. A total of 50 participants were initially recruited for the recall group. Six participants were excluded because they withdrew before the end of the experiment, and one participant was excluded due to excessive head motion during the fMRI scan. The final sample for the recall group consisted of 43 participants (27 females and 16 males, mean age 22 ± 2 years). The control group consisted of 57 participants (29 females and 28 males, mean age 22 ± 2 years). There were no significant differences in gender or age between the two groups (*ps* > 0.11). The 57 participants in the control group of this study were the same 57 participants included in Experiment 2 of our previously published paper [46]. In our previous study, we examined the misinformation effect on intra-subject (within-individual) neural representations using data from these 57 participants. In this study, we reanalyzed their neural data to examine inter-subject (between-individual) neural representations when viewing original events. The sample size was based on previous studies on similar topics [41,43–47]. Participants were right-handed Chinese college students with normal vision and speech and no history of psychiatric or neurological disorders. They were screened to ensure that they had not been exposed to any materials related to this study.

### Stimuli

Stimuli were developed for eight events, including images, written narratives, and memory tests. These eight events are about stealing, scalper, gamble, robbery, bullying, scam, loan shark, and family conflicts. Each event has a title (e.g., a man stole a girl’s phone on the street). Images of the first three events were taken by the authors, while those of the last five events were captured from television videos. For each event, 50 color images and 50 corresponding narrative sentences were used in the original-event stage and the post-event misinformation stage, respectively. Each event consisted of 12 critical scenes and 38 noncritical scenes. To achieve a balanced design, we created two versions of the image for each critical scene (Fig 1). Each participant viewed one of two versions of critical scenes for each of eight events, which were randomly assigned. For example, Participant 1 viewed the following versions of the eight events: A, B, B, A, A, B, A, B, while Participant 2 viewed: B, B, A, B, A, A, A, B. In the end, 15, 25, 26, 18, 19, 25, 16, and 23 of the 43 participants in the recall group viewed version A of each of the eight events during the original-event stage, while the remaining participants (i.e., 28, 18, 17, 25, 24, 18, 27, and 20 participants) viewed version B. Of the 57 participants in the control group, 23, 34, 33, 27, 28, 30, 20, and 31 viewed version A, while 34, 23, 24, 30, 29, 27, 37, and 26 participants viewed version B. For critical scenes, the narratives at the post-event stage contained erroneous details (i.e., the details differed between the original-event stage and the post-event stage). For noncritical scenes, the narratives during the post-event stage were accurate (i.e., the details were the same as those at the original-event stage). Please refer to the three columns on the left side of S5 Table for specific examples. To increase the credibility of the narrative, each event began with two noncritical scenes, and no two critical scenes in each event would appear consecutively. The criteria used to select the details of critical scenes were based on the methods employed in previous studies [45,78]. They primarily fell into the following five categories: object (e.g., color or shape), text (e.g., letter or word), number (e.g., digit or quantity), agent (e.g., body part or action), and location (e.g., location or relative position). The number of scenes belonging to these five categories was 29, 29, 20, 11, and 7, respectively. These details of critical scenes represent both central and peripheral aspects of the plot. See the Supporting Information S1 Text for details. In terms of memorability, the two versions of the details of each critical scene are matched. This is evident in the behavioral data from the initial free recall test of the recall group. For the 96 critical scenes, an average of 20% of participants who viewed version A were able to freely recall details from version A, while an average of 19% of participants who viewed version B were able to freely recall details from version B. There was no significant difference in memorability between these two versions (*t*(190) = 0.35, *p* = 0.728).

### Experimental procedure

The experiment for the recall group consisted of four stages, namely, viewing the original events, initial free recall, reading narratives with misinformation, and final free recall of original events (Fig 3A). This procedure is a modified version of the classic three-stage misinformation paradigm (i.e., viewing the original events, reading narratives with misinformation, and recognition tests of original events), which was employed as the control group [46]. Both groups of participants underwent the same experimental procedure inside the fMRI scanner to encode the original events. The procedure for the recall group differed from the control group in only two key aspects. First, participants in the recall group were informed prior to the encoding of original events that they would later take spoken free recall tests. By contrast, participants in the control group were told they would take memory tests without being informed of the specific type. This study aimed to investigate the effect of participants’ expectations about the type of test they would take on their encoding process, by comparing the neural representations of the recall and control groups when encoding original events. Secondly, participants in the recall group completed two free recall tests—one before and one after encoding the misinformation—whereas the control group employed recognition tests in the end. This design aimed to examine misinformation-induced false memories during the final recall, while excluding spontaneous false memories that might have emerged during the initial recall.

In the recall group, participants completed all four stages in the fMRI scanner. Each stage lasted ~30 min and consisted of eight events spread over four runs (i.e., two events per run). The order of presentation or recall of these 8 events was randomized across participants in each of these stages to avoid the potential sequential effect between events. There was a 10-min interval between the original-event stage and the initial free recall stage, a 3-hour interval between the initial free recall stage and the post-event misinformation stage, and a 10-min interval between the post-event misinformation stage and the final free recall stage. Participants were asked to return to the lab on time and not to discuss the events with others. To standardize the experimental procedure and minimize experimenter’s intervention, all instructions were prerecorded and played back by the researchers.

#### The original-event stage.

Participants saw 50 images for each of the eight events. Before encoding, they were told to remember each image depicting each scene of the event. The recall group was informed about the upcoming free recall tests, while the control group was informed about the upcoming memory tests. Each image was shown for 3.5 s, with each image being presented only once. A black fixation cross was presented for 0.5 s between images. To prevent participants from falling asleep during the fMRI scan, a red fixation cross was randomly displayed before two to four of the 38 noncritical images in each event. Participants were instructed to press a button as quickly and accurately as possible with their right index finger, when they observed the red fixation cross instead of the black one. At the beginning and end of each of the eight events, a 3.5-s visual cue labeled with the event title was displayed.

#### The initial free recall stage.

Participants in the recall group were instructed to give a verbal account of what they could remember about the original events, providing as much detail as possible. They were encouraged to speak for a total of 3 min on each of the eight events. To assist the participants in recalling the event, a visual cue with the title of the event was displayed for 10 s prior to the recall of each event. Recall began with the appearance of a white dot on the gray screen, which remained visible for 2 min and 50 s. This was followed by a red dot that remained visible for 10 s, indicating that the recall of the event was coming to an end. A 4-s visual cue with the title of the event was then displayed, indicating the official end point of the event recall. Participants were then asked to recall the next event until they had completed the recall of all eight events. The order of the events that each participant was asked to recall was randomized.

#### The post-event misinformation stage.

Participants read 50 sentences for each of the eight events. They were told that these sentences were narrated by another eyewitness, but were not warned about possible discrepancies between the images and the narratives. Each sentence was presented on a horizontal line in the center of the screen. Post-event narrative with misinformation was presented in the same manner as in the original-event stage (e.g., start/end cues, fixation cross, and stimulus presentation durations).

#### The final free recall stage.

Participants in the recall group were asked to recall what they remembered about the original images they initially saw in the original events. The words “original images” were printed in red ink and highlighted in the instructions. This procedure was identical to the initial free recall stage. At the end of the study, participants received a debriefing.

### Behavioral analysis

#### Recall scoring.

Participants’ free recall was transcribed and automatically timestamped using the video editing software CapCut (see S1 Text). The transcriptions were then manually checked and coded by two raters independently (see below for inter-rater reliability). Although participants saw the title of each event before being asked to recall it, it was still possible to incorrectly recall another event that did not match the title. Of the 43 participants who recalled the eight events twice, only two participants recalled an incorrect event for the given title in the final recall. These participants’ recall data for the event were recoded as missing data. To determine the correspondence between the content of the participant’s recall and the images of the events, the raters assigned the content of the participant’s recall to the most appropriate scene from the 50 images of each event. The recalled content was then labeled with a scene number ranging from 1 to 50. It should be noted that the recall needs to contain only the main plots in order to align with the scene, and it may or may not include critical details. However, if the recalled content was merely a repetition of the event title or was irrelevant (e.g., “This event is interesting.” or “He may be a student.”), it was labeled as 0. Of all the sentences recalled for the 8 events for the 43 participants, a total of 130 sentences were marked as 0 for the initial recall, and a total of 75 sentences were marked as 0 for the final recall. In the behavioral analysis, we did not use the data labeled as 0. In the neural analysis, we used it as a nuisance variable when we performed the single-trial modeling. After completing this labeling process, we determined the timestamps for the beginning (i.e., onset) and duration of these recalled contents. To assess the accuracy of details recalled for each event, we used an adapted version of the scoring method used in the autobiographical memory interviews [19,79,80].

To further assess the recall of each of the 96 critical scenes, the raters classified them into one of the following five mutually exclusive categories. These included original (i.e., recall of the critical detail from the original-event), misinformation (i.e., recall of the critical detail from the misinformation), foil (i.e., recall of the critical detail but from neither the original-event nor the misinformation), no-critical-detail (i.e., recall of the scene without the critical detail), and unrecalled (i.e., no recall of this scene at all) (Fig 3B). The counts by category were converted into percentages, out of the total number of 96 scenes/details (one detail per scene), for the initial and final recall separately.

As an example, a participant saw the man hiding behind a lamp post in the original-event and later read the post-event misinformation that the man was hiding behind a tree trunk. If this participant recalled “the man was hiding behind a lamp post”, it was coded as original; “the man was hiding behind a tree trunk” coded as misinformation; “the man was hiding behind a car” coded as foil; and “the man was hiding somewhere” coded as no-critical-detail. If the participant did not recall the scene, it was coded as “unrecalled”. Two raters independently coded the recalls. The inter-rater reliability was high (Kappa = 0.95).

#### Critical scenes were reported in the initial and final free recall tests.

A two-way repeated measures ANOVA with post hoc analyses was used to compare differences in the percentages of four types of recall performance on critical scenes (i.e., original, misinformation, foil, and no-critical-detail) between two recall stages (i.e., the initial and final recall). Next, recall performance on critical scenes in the final recall was further refined as persistent true memory and misinformation-induced false memory. Persistent true memory was defined as accurately recalling the detail of the original-event in both the initial and final recalls of a critical scene. Misinformation-induced false memory was defined as recalling the misinformation in the final recall but not in the initial recall of a critical scene. In other words, if a participant reported a piece of misinformation spontaneously during the initial recall (before the post-event misinformation stage), it would not be considered misinformation-induced false memory. Other types of recall (e.g., persistent unspecific memory) did not contain original or misinformation details, and were therefore not included in the following neural analyses. Using these refined indices, we also examined shared memories (i.e., pairs of participants who showed the same type of recall after seeing the same or different versions of critical scenes). It is important to note that shared false memories refer to shared misinformation-induced false memories (recalled only in the final recall). Whereas shared true memories are shared persistent true memories (recalled in both the initial and final recalls). Two-tailed *p* values were reported for the behavioral analysis.

### fMRI data collection and analysis

#### MRI acquisition.

Brain image data were acquired by a 3.0 T Siemens Prisma MRI scanner with a 64-channel head coil at the Brain Imaging Center of Beijing Normal University. An MRI-compatible microphone (Optoacoustics FOMRI-III) with bidirectional noise-canceling was affixed to the head coil, and the receiver was placed over the participant’s mouth. The high-resolution functional images were acquired using a simultaneous multi-slice EPI sequence (TR/TE/θ = 2,000 ms/34 ms/90°; FOV = 200 mm × 200 mm; matrix = 100 × 100; in-plane resolution = 2 × 2 mm; slice thickness = 2 mm; multi-band acceleration factor = 3; slices = 72). The high-resolution structural image was acquired for the whole-brain using a T1-weighted MPRAGE sequence (TR/TE/θ = 2,530 ms/2.27 ms/ 7°; FOV = 256 mm × 256 mm; matrix = 256 × 256; slice thickness = 1 mm). The field map was acquired using a Gradient Echo sequence to correct for magnetic field distortions (TR = 750 ms; θ = 60°; TE1/TE2 = 5.20 ms/7.66 ms; FOV = 200 mm × 200 mm; matrix = 100 × 100; slice thickness = 2 mm; slices = 72).

#### Image preprocessing.

Imaging data preprocessing was performed using fMRIPrep v23.1.4. The structural images were corrected for intensity nonuniformity using N4BiasFieldCorrection, and skull-stripping was performed using antsBrainExtraction.sh with OASIS30ANTs as the target template. Spatial normalization to the MNI152NLin2009cAsym template was done by nonlinear registration using antsRegistration. The field map was co-registered to the reference volume and converted to a displacement map for each functional run. The BOLD reference was co-registered to the corresponding structural data using boundary-based registration with six degrees of freedom. Slice time correction was applied to each functional run using 3dTshift (AFNI), which was then resampled to its native space using a single composite transform to correct for head motion and susceptibility distortions. Frame-wise displacement was computed for each functional run using the Nipype implementation. The data were then temporally filtered using a nonlinear high-pass filter with a 100-s cutoff. To improve the signal-to-noise ratio, we used data smoothed by a 2-mm full-width half-maximum Gaussian kernel for the neural pattern similarity analysis.

#### Single-trial estimation.

Single-trial estimation was used to increase the sensitivity of multivariate analyses [81]. The General Linear Model (GLM; FSL’s FILM module version 6.00) was used to compute the *t*-statistic map for each of the stimulus trials (i.e., 50 scenes × 8 events × 2 encoding stages × 43 participants = 34,400 trials) at the original-event and post-event misinformation encoding stages (3.5 s per trial) and the recall trials (i.e., a total of 15,847 recalled sentences, which is 46% of the total 34,400 possible sentences). The durations of these recalled trials are shown in S8 Table (7 ± 4 s per trial on average). In this single-trial model, the presentation of each trial was modeled as an impulse and convolved with a double-gamma hemodynamic response function. Nuisance variables in each single-trial model included six motion parameters and frame-wise displacement, as well as the red fixation during the original-event and post-event stages. The least squares single method was used to obtain reliable estimates of single-trial responses. The estimated *t-*value was obtained for each trial for each subject, and was used to calculate the neural pattern similarity in the following statistical analysis.

#### Inter-subject similarity between all participants when encoding original events.

To investigate whether participants’ expectations about test types influence their shared encoding of event details, we compared the detail-specific representations shared by participants in the recall group with those shared by participants in the control group during the encoding of original events (Fig 2A). First, we examined which brain regions in each group were involved in the shared encoding of event details. Since this analysis involved all critical scenes viewed by all participants during the encoding stage, we performed participant-level averaging of the neural metrics. In other words, each participant had two inter-subject scene-specific similarity values. One is the average value calculated based on the similarities between that participant and all the other participants in their group who watched the same version (i.e., same version [C minus N]). C indicates the inter-subject similarity for the corresponding scene, and N indicates the average inter-subject similarity for noncorresponding scenes within the same event. The second is the average value calculated based on the similarities between that participant and all the other participants in their group who watched different versions (i.e., different versions [C minus N]). The paired *t* test was used to compare the inter-subject scene-specific neural similarity between the same and different versions within each group (i.e., same version [C minus N] versus different versions [C minus N]).

Next, we examined which brain regions involved in the shared encoding of event details were affected by test expectations. This was achieved by comparing the two groups. Each participant had an inter-subject detail-specific similarity value (i.e., same version [C minus N] minus different versions [C minus N]). The independent *t* test was used to examine the inter-subject detail-specific neural similarity between the recall and control groups (i.e., recall versus control).

#### Inter-subject similarity during encoding and recall for shared memories.

To examine shared neural representations during encoding or recall for shared memories, we computed the similarity of neural activity patterns between participants who formed either shared true or false memories after viewing the same or different versions of critical scenes (Figs 4A and 5A). This was done separately for each encoding and recall stage. We examined four stages for shared true memories and three stages for shared false memories. This is because false memories induced by misinformation only emerged in the final recall, whereas persistent true memories appear in both the initial and final recall.

Scene-specific representation was indicated by greater inter-subject similarity for the corresponding (C) scene than the average similarity value for the noncorresponding (N) scenes in the same event (i.e., C > N). These analyses were conducted at the trial level, that is, between each pair of participants who shared a memory of a specific scene. To obtain scene-specific values for each trial, these similarity indices were then adjusted by the values of the corresponding scene minus the average of the remaining noncorresponding scenes in the same event. The data were calculated for individuals who shared memories after viewing the same version or different versions of critical scenes, separately. Detail-specific representation was indicated by greater inter-subject scene-specific similarity for the same version than that for different versions (i.e., same version [C minus N] > different versions [C minus N]). The mixed-effects model was employed to examine the inter-subject scene-specific and detail-specific neural similarity during each stage for each shared memory type. The model incorporated both participant identity and stimulus identity as random effects.

We present an example to explain how we calculated the neural pattern similarity among three participants when viewing one of the original events, who later formed shared true memories of a specific detail (S2 Fig). First, we identified the neural activity patterns of the three participants in the brain region of interest while they encoded the original images of the event. Then, we calculated the similarity of neural patterns between participants viewing the same version of the corresponding scene. For example, let’s consider the similarity between participants R and S when viewing the 50th scene of this event. Specifically, we calculated the Pearson correlation coefficient between these two participants’ neural activity patterns and transformed it into a Fisher’s Z-score. As a baseline, we also calculated the similarity of neural patterns between these participants for the noncorresponding scenes in the same event. This was achieved by averaging 49 Fisher’s Z scores, reflecting the inter-subject similarity between one participant viewing a critical scene and another participant viewing the other 49 scenes in the same event (e.g., the similarity between participant R viewing the 50th scene and participant S viewing the 1st scene of this event). Next, we calculated the similarity of neural patterns between participants who shared true memories after viewing different versions of the corresponding scene (e.g., the similarity between participants R and T when viewing the 50th scene of this event). Finally, we calculated the similarity of neural patterns between these participants for the noncorresponding scenes in the same event (e.g., the similarity between participant R viewing the 50th scene and participant T viewing the 1st scene of this event).

Using the same method, we calculated the similarity of neural patterns between participants who formed shared true memories during either the encoding of misinformation or the free recall stage (Fig 4A). The only difference was in the way noncorresponding scenes were calculated during free recall. All participants viewed 50 images and 50 sentences for each event during encoding. However, the number of scenes that each participant recalled for each event varied during free recall. Therefore, for the noncorresponding scenes during recall, we averaged the results from the remaining scenes (except for the corresponding scene) that were recalled by another participant. The same procedure was used to calculate the similarity of neural patterns between participants who formed shared false memories (Fig 5A).

#### Regions of interest (ROI).

***Cortical regions.*** We used a cortical parcellation atlas based on fMRI functional connectivity patterns (i.e., Schaefer2018_400Parcels_17Networks) for whole-brain pattern-based analyses. Specifically, the cortical surface was divided into 400 parcels. Among all participants in the recall or control group, the paired *t* test was used to compare the inter-subject scene-specific neural similarity between the same and different versions (i.e., same versus different versions). Finally, the independent *t* test was then used to examine the inter-subject detail-specific neural similarity between the recall and control groups (i.e., recall versus control). In addition to parametric *t*-tests, permutation tests were conducted to further assess the robustness of the results. Null distributions were generated from 10,000 permutations by randomly shuffling version labels or group labels, respectively. Two-tailed *p*-values were calculated as the proportion of permutations in which the absolute *t*-value was greater than or equal to the observed absolute *t*-value. The false discovery rate (FDR) method was used to correct for multiple comparisons across all 400 parcels for both parametric and permutation-based analyses.

Among participants with shared true or false memories, the linear mixed-effects model was first used to examine whether scene-specific representations were present in each of these cortical regions during the encoding or recall stage. The model was specified as:

*intersubject similarity ~ specificity + (1| paired subject) + (1| event) + (1| stimulus),*

where specificity was a two-level factor (corresponding versus noncorresponding scenes), and paired subject denoted the concatenated identities (IDs) of the two participants whose neural pattern was used to compute inter-subject similarity.

Next, we used the second mixed-effects model to examine whether these cortical regions also exhibited detail-specific representations during the encoding or recall stage among participants with shared true or false memories. The model was specified as:

*intersubject similarity ~ version + (1| paired subject) + (1| event) + (1| stimulus),*

where version was a two-level factor (same versus different versions). To further assess the robustness of the results, permutation tests were conducted in parallel with the parametric linear mixed-effects analyses. To assess the significance of the fixed effect, null distribution was generated from 10,000 permutations by randomly shuffling version labels across paired subject IDs within each event. Two-tailed *p*-values were calculated as the proportion of permutations in which the absolute *t* value was greater than or equal to the observed absolute *t* value.

Following the approach described in previous studies [82–84], we applied the FDR method to correct *p*-values from the entire cortical surface for multiple comparisons across all 400 parcels. Taking the neural representation of a specific type of shared memory at a certain stage as an example, we first performed the multiple comparison correction on scene-specific results across 400 cortical regions to identify regions that remained significant after the FDR correction (same version: corresponding > noncorresponding scenes). Next, we applied the multiple comparison correction to detail-specific results across these 400 cortical regions to identify cortical regions that remained significant after the FDR correction (corresponding minus noncorresponding scenes: same > different versions). Finally, we identified cortical regions that were significant after the FDR correction in both analyses above, which were then considered to possess detail-specific neural representations.

***Hippocampus.*** The bilateral hippocampus in the MNI space was used for the inter-subject analysis. A mixed-design ANOVA was used to examine whether the recall group exhibited stronger detail-specific representations in the hippocampus when encoding original events compared to the control group. Two-tailed *p* values were reported for the analysis of the hippocampus. Among participants with shared true or false memories, the linear mixed-effects model was used to examine the presence of detail-specific representations in the hippocampus during each stage. The related content is reported in S12 Table.

## Supporting information

S1 Data Supporting data underlying Figures (Figs 2–5 and S1) and Tables (S4, S6–S12 Tables). (XLSX)

S1 Fig Recall of critical scenes. Percentages of original, misinformation, foil, and no-critical-detail in the initial and final recall. The misinformation effect was evidenced by a greater increase in the amount of misinformation than foil from the initial to the final recall. Error bars show the standard error of the mean. Each white dot represents one participant. The performance of the same participant in the initial and final recall is connected by a line. The underlying numerical data for this figure are provided in S1 Data.
(TIF)

S2 Fig An example to explain the calculation of the neural pattern similarity among three participants when viewing one of the original events, who later formed shared true memories of a specific detail. First, we identified the neural activity patterns of the three participants in the brain region of interest while they encoded the original images of the event. Then, we calculated the similarity of neural patterns between participants viewing the same version of the corresponding scene (C). For example, let’s consider the similarity between participants R and S when viewing the 50th scene of this event (connected by a black solid curve labeled “C”). Specifically, we calculated the Pearson correlation coefficient between these two participants’ neural activity patterns and transformed it into a Fisher’s Z-score. As a baseline, we also calculated the similarity of neural patterns between these participants for the noncorresponding scenes in the same event (N). This was achieved by averaging 49 Fisher’s Z scores, reflecting the inter-subject similarity between one participant viewing a critical scene and another participant viewing the other 49 scenes in the same event (e.g., the similarity between participant R viewing the 50th scene and participant S viewing the 1st scene of this event, connected by a gray solid curve labeled “N”). Next, we calculated the similarity of neural patterns between participants who shared true memories after viewing different versions of the corresponding scene (e.g., the similarity between participants R and T when viewing the 50th scene of this event, connected by a black dashed curve labeled “C”). Finally, we calculated the similarity of neural patterns between these participants for the noncorresponding scenes in the same event (e.g., the similarity between participant R viewing the 50th scene and participant T viewing the 1st scene of this event, connected by a gray dashed curve labeled “N”).
(TIF)

S1 Table Brain regions that showed detail-specific representations were shared by all participants in the recall group when encoding original events. (PDF)

S2 Table Brain regions that showed detail-specific representations were shared by all participants in the control group when encoding original events. (PDF)

S3 Table Brain regions that showed stronger detail-specific representations in the recall group than in the control group when encoding original events. (PDF)

S4 Table Inter-subject similarity in brain regions that showed stronger detail-specific representations in the recall group than in the control group when they encoded original events (Mean ± SD). The underlying numerical data for this table are provided in S1 Data.
(PDF)

S5 Table Examples and recall type for critical and noncritical scenes. (PDF)

S6 Table Percentages of critical scenes by type of recall (%). The underlying numerical data for this table are provided in S1 Data.
(PDF)

S7 Table Percentages of critical scenes for persistent and nonpersistent memories in the final recall (%). The underlying numerical data for this table are provided in S1 Data.
(PDF)

S8 Table The duration in each recalled sentence for critical scenes (Mean ± SD). The underlying numerical data for this table are provided in S1 Data.
(PDF)

S9 Table Percentages of noncritical scenes by type of recall (%). The underlying numerical data for this table are provided in S1 Data.
(PDF)

S10 Table Inter-subject neural pattern similarity in brain regions that showed detail-specific representations between people with shared true memories (Mean ± SD). The underlying numerical data for this table are provided in S1 Data.
(PDF)

S11 Table Inter-subject neural pattern similarity in brain regions that showed detail-specific representations between people with shared false memories (Mean ± SD). The underlying numerical data for this table are provided in S1 Data.
(PDF)

S12 Table Inter-subject neural pattern similarity in the hippocampus (Mean ± SD). The underlying numerical data for this table are provided in S1 Data.
(PDF)

S1 Text An example of one participant’s free recall of one event with scoring. (PDF)

## Abbreviations

FDR: false discovery rate
GLM: General Linear Model
LFEF: left frontal eye fields
LMTG: left middle temporal gyrus
LMCC: left middle cingulate cortex
LPCC: left posterior cingulate cortex
RAG: right angular gyrus
RMOC: right medial occipital cortex
ROI: regions of interest.
