# Supplementary figures and images for: Shared memories of event details in the human brain are altered by misinformation and test expectations
Source: PLoS Biol. 2026 Jul 6;24(7):e3003886. doi: 10.1371/journal.pbio.3003886 (PMC13336189; doi:10.1371/journal.pbio.3003886)

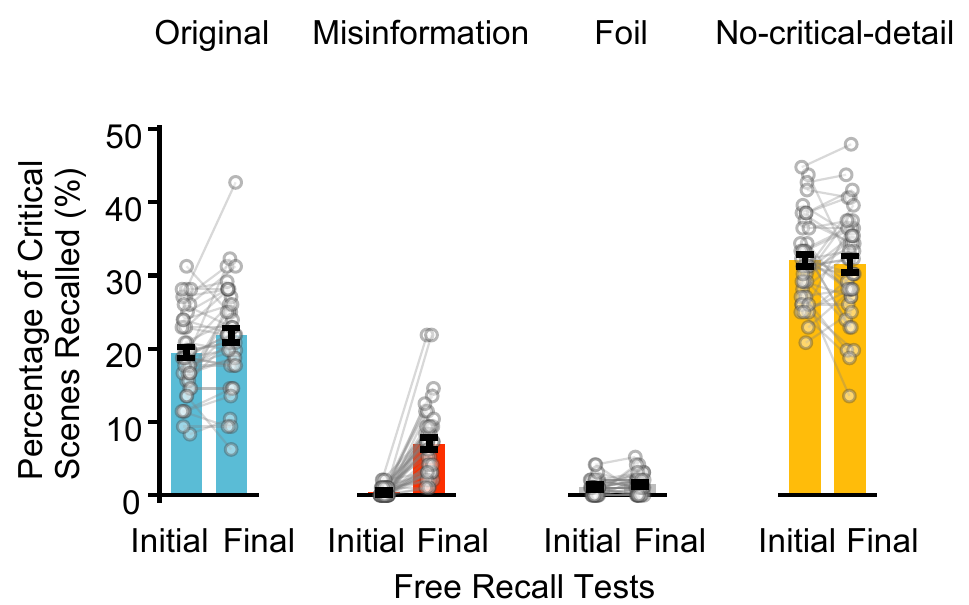

Supplement: S1 Fig — Percentages of original, misinformation, foil, and no-critical-detail in the initial and final recall. The misinformation effect was evidenced by a greater increase in the amount of misinformation than foil from the initial to the final recall. Error bars show the standard error of the mean. Each white dot represents one participant. The performance of the same participant in the initial and final recall is connected by a line. The underlying numerical data for this figure are provided in S1 Data. (TIF) [file pbio.3003886.s002.tif]

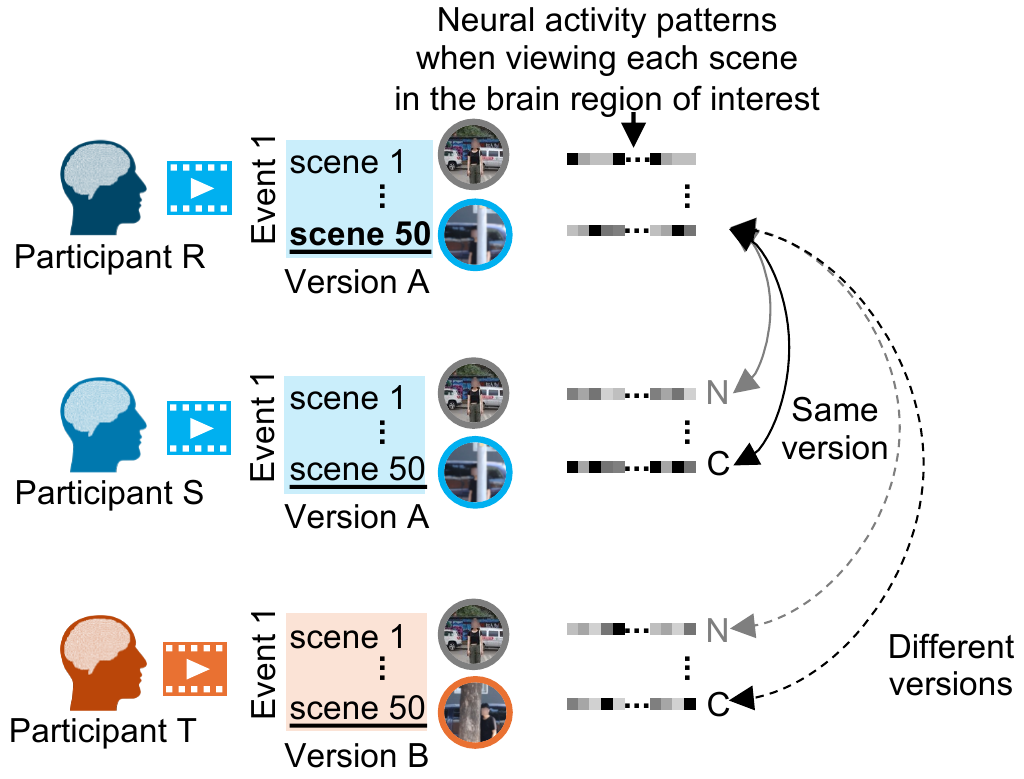

Supplement: S2 Fig — First, we identified the neural activity patterns of the three participants in the brain region of interest while they encoded the original images of the event. Then, we calculated the similarity of neural patterns between participants viewing the same version of the corresponding scene (C). For example, let’s consider the similarity between participants R and S when viewing the 50th scene of this event (connected by a black solid curve labeled “C”). Specifically, we calculated the Pearson correlation coefficient between these two participants’ neural activity patterns and transformed it into a Fisher’s Z-score. As a baseline, we also calculated the similarity of neural patterns between these participants for the noncorresponding scenes in the same event (N). This was achieved by averaging 49 Fisher’s Z scores, reflecting the inter-subject similarity between one participant viewing a critical scene and another participant viewing the other 49 scenes in the same event (e.g., the similarity between participant R viewing the 50th scene and participant S viewing the 1st scene of this event, connected by a gray solid curve labeled “N”). Next, we calculated the similarity of neural patterns between participants who shared true memories after viewing different versions of the corresponding scene (e.g., the similarity between participants R and T when viewing the 50th scene of this event, connected by a black dashed curve labeled “C”). Finally, we calculated the similarity of neural patterns between these participants for the noncorresponding scenes in the same event (e.g., the similarity between participant R viewing the 50th scene and participant T viewing the 1st scene of this event, connected by a gray dashed curve labeled “N”). (TIF) [file pbio.3003886.s003.tif]
