# Supplementary material for: Shared memories of event details in the human brain are altered by misinformation and test expectations
Source: PLoS Biol. 2026 Jul 6;24(7):e3003886. doi: 10.1371/journal.pbio.3003886 (PMC13336189; doi:10.1371/journal.pbio.3003886)
Supplement: S1 Table — (PDF) [file pbio.3003886.s004.pdf]

S1 Table. Brain regions that showed detail-specific representations shared by all participants in the recall group when encoding original events.

Label	Region	x	y	z	T test FDR-corrected *p*-values	Permutation FDR-corrected *p*-values
7	Left medial occipital cortex	-8	-98	-8	0.0221	0.0229
207	Right medial occipital cortex	8	-92	-2	0.0008	0.0133
212	Right lateral occipital cortex	28	-88	20	0.0200	0.0229
398	Right superior temporal gyrus	64	-34	10	0.0200	0.0133
83	Left frontal eye fields	-26	0	56	0.0200	0.0229
133	Left middle cingulate cortex	-4	6	28	0.0200	0.0250
158	Left posterior cingulate cortex	-2	-16	38	0.0080	0.0133
