# Supplementary material for: Shared memories of event details in the human brain are altered by misinformation and test expectations
Source: PLoS Biol. 2026 Jul 6;24(7):e3003886. doi: 10.1371/journal.pbio.3003886 (PMC13336189; doi:10.1371/journal.pbio.3003886)
Supplement: S2 Table — (PDF) [file pbio.3003886.s005.pdf]

S2 Table. Brain regions that showed detail-specific representations shared by all participants in the control group when encoding original events.

Label	Region	x	y	z	T test FDR-corrected *p*-values	Permutation FDR-corrected *p*-values
18	Left medial occipital cortex	-4	-88	2	0.0052	0.0133
1	Left fusiform gyrus	-36	-62	-16	0.0243	0.0514
13	Left lingual gyrus	-24	-54	-8	0.0009	0.0133
205	Right ventrolateral occipital cortex	42	-84	-12	0.0315	0.0480
324	Right inferior temporal gyrus	50	-28	-26	0.0211	0.0300
330	Right lateral prefrontal cortex	50	30	18	0.0459	0.0514
