# Supplementary material for: Shared memories of event details in the human brain are altered by misinformation and test expectations
Source: PLoS Biol. 2026 Jul 6;24(7):e3003886. doi: 10.1371/journal.pbio.3003886 (PMC13336189; doi:10.1371/journal.pbio.3003886)
Supplement: S3 Table — (PDF) [file pbio.3003886.s006.pdf]

S3 Table. Brain regions that showed stronger detail-specific representations in the recall group than in the control group when encoding original events.

Label	Region	x	y	z	T test FDR-corrected *p*-values	Permutation FDR-corrected *p*-values
83	Left frontal eye fields	-26	0	56	0.0030	0.0100
158	Left posterior cingulate cortex	-2	-16	38	0.0025	0.0100
133	Left middle cingulate cortex	-4	6	28	0.0167	0.0100
207	Right medial occipital cortex	8	-92	-2	0.0167	0.0200
