# Supplementary material for: Shared memories of event details in the human brain are altered by misinformation and test expectations
Source: PLoS Biol. 2026 Jul 6;24(7):e3003886. doi: 10.1371/journal.pbio.3003886 (PMC13336189; doi:10.1371/journal.pbio.3003886)
Supplement: S4 Table — The underlying numerical data for this table are provided in S1 Data. (PDF) [file pbio.3003886.s007.pdf]

S4 Table. Inter-subject similarity in brain regions that showed stronger detail-specific representations in the recall group than in the control group when they encoded original events (Mean ± SD). The underlying numerical data for this figure are provided in S1 Data.

Region (label)	Group	Same version		Different versions	
		Corresponding scene	Non-corresponding scenes	Corresponding scene	Non-corresponding scenes
LEFE	Recall	0.0116 ± 0.0089	0.0067 ± 0.0077	0.0084 ± 0.0088	0.0052 ± 0.0075
(83)	Control	0.0078 ± 0.0041	0.0036 ± 0.0033	0.0090 ± 0.0039	0.0042 ± 0.0033
LPCC	Recall	0.0163 ± 0.0094	0.0112 ± 0.0075	0.0128 ± 0.0077	0.0100 ± 0.0070
(158)	Control	0.0078 ± 0.0049	0.0055 ± 0.0042	0.0081 ± 0.0054	0.0053 ± 0.0046
LMCC	Recall	0.0047 ± 0.0047	0.0018 ± 0.0041	0.0019 ± 0.0042	0.0013 ± 0.0042
(133)	Control	0.0039 ± 0.0036	0.0025 ± 0.0029	0.0044 ± 0.0040	0.0024 ± 0.0031
RMOC	Recall	0.1419 ± 0.0362	0.1038 ± 0.0305	0.1375 ± 0.0355	0.1022 ± 0.0305
(207)	Control	0.1419 ± 0.0316	0.1098 ± 0.0282	0.1420 ± 0.0322	0.1104 ± 0.0291

Note：left frontal eye fields (LEFE)，left posterior cingulate cortex (LPCC), Left middle cingulate cortex (LMCC), and right medial occipital cortex (RMOC).
