# Supplementary material for: Shared memories of event details in the human brain are altered by misinformation and test expectations
Source: PLoS Biol. 2026 Jul 6;24(7):e3003886. doi: 10.1371/journal.pbio.3003886 (PMC13336189; doi:10.1371/journal.pbio.3003886)
Supplement: S5 Table — (PDF) [file pbio.3003886.s008.pdf]

S5 Table. Examples and recall type for critical and non-critical scenes.

	Original-event stage	Post-event (misinformation) stage	Recall type
Critical scenes	96 images for critical scenes (e.g., a man hid behind a lamp post)	96 sentences for narratives with misinformation (e.g., a man hid behind a tree trunk)	**original** (e.g., a man hid behind a lamp post)
			**misinformation** (e.g., a man hid behind a tree trunk)
			**foil** (e.g., a man hid behind a car)
			**no-critical-detail** (e.g., a man hid somewhere)
			unrecalled
Non-critical scenes	304 images for non-critical scenes (e.g., a girl walks past a soda can)	304 sentences for narratives with accurate information (e.g., a girl walks past a soda can)	correct (e.g., a girl walks past a soda can)
			incorrect (e.g., a girl walks past a plastic bag)
			unrecalled

Note: Critical scene: If a participant saw an image of a man hiding behind a lamp post during the original-event stage, then he or she read a narrative about a man hiding behind a tree trunk during the post-event stage. Non-critical scene: If a participant saw an image of a girl walking past a soda can during the original-event stage, then he or she read a narrative about a girl walking past a soda can during the post-event stage.
