# Supplementary material for: Shared memories of event details in the human brain are altered by misinformation and test expectations
Source: PLoS Biol. 2026 Jul 6;24(7):e3003886. doi: 10.1371/journal.pbio.3003886 (PMC13336189; doi:10.1371/journal.pbio.3003886)
Supplement: S6 Table — The underlying numerical data for this table are provided in S1 Data. (PDF) [file pbio.3003886.s009.pdf]

S6 Table. Percentages of critical scenes by type of recall (%). The underlying numerical data for this figure are provided in S1 Data.

	Initial recall	Final recall	Initial vs. final recall		
	(Mean ± SD)	(Mean ± SD)	*t*	*p*	Cohen's *d*
**Original**	19.48 ± 5.53	21.85 ± 6.95	3.46	$1e^{-3}$	0.36
**Misinformation**	0.44 ± 0.65	7.05 ± 4.85	8.97	$3e^{-11}$	1.80
**Foil**	1.14 ± 1.06	1.48 ± 1.29	1.53	0.13	0.28
**No-critical-detail**	32.05 ± 5.72	31.54 ± 7.17	-0.59	0.56	-0.08
Unrecalled	46.90 ± 7.43	38.08 ± 7.21	-14.44	$7e^{-18}$	-1.18

Note: SD: standard deviation. It is important to note that the misinformation reported in the initial recall (0.44%) was not caused by reading post-event narratives with misinformation, because the post-event misinformation was presented after the initial recall. Instead, it came from spontaneous recall of erroneous details that happened to be the same as the misinformation about to be presented. The 2 × 4 repeated measures ANOVA (excluding the unrecalled) showed significant main effects of recall stage (initial and final) ($F(1, 42) = 208.50$, $p = 2e^{-16}$, $\eta^2_p = 0.83$), and recall type (original, misinformation, foil, and no-critical-detail) ($F(3, 126) = 428.00$, $p = 1e^{-16}$, $\eta^2_p = 0.91$), as well as their interaction on the percentage of critical scenes ($F(3, 126) = 17.58$, $p = 1e^{-9}$, $\eta^2_p = 0.30$). Post hoc analyses showed that the recall of original critical details and misinformation increased from the initial to the final recall ($ps < 1e^{-3}$), but that of foils and no-critical-details did not ($ps > 0.13$). More important, the increase in the recall of misinformation from the initial to the final recall was greater than that in the other three types of recall.
