# Supplementary material for: Shared memories of event details in the human brain are altered by misinformation and test expectations
Source: PLoS Biol. 2026 Jul 6;24(7):e3003886. doi: 10.1371/journal.pbio.3003886 (PMC13336189; doi:10.1371/journal.pbio.3003886)
Supplement: S7 Table — The underlying numerical data for this table are provided in S1 Data. (PDF) [file pbio.3003886.s010.pdf]

S7 Table. Percentages of critical scenes for persistent and non-persistent memories in the final recall (%). The underlying numerical data for this figure are provided in S1 Data.

Recall type defined based on changes from the initial to the final recall (in parentheses)	Mean ± SD
**Persistent true** (original → original)	**16.59 ± 5.24**
Non-persistent true	5.26 ± 3.66
(no-critical-detail → original)	(2.37 ± 2.30)
(unrecalled → original)	(2.54 ± 1.71)
(foil → original)	(0.27 ± 0.51)
(misinformation → original)	(0.07 ± 0.27)
**Misinformation-induced false**	**6.78 ± 4.83**
<u>(original → misinformation)</u>	<u>(0.92 ± 1.07)</u>
<u>(foil → misinformation)</u>	<u>(0.15 ± 0.37)</u>
<u>(no-critical-detail → misinformation)</u>	<u>(2.96 ± 2.41)</u>
(unrecalled → misinformation)	(2.76 ± 2.53)
Persistent false (misinformation → misinformation)	0.27 ± 0.46
Persistent unspecific (no-critical-detail → no-critical-detail)	20.37 ± 6.21
Non-persistent unspecific	11.17 ± 3.09

Note: (1) Persistent true memory: For example, if a participant recalled 7 critical scenes (1, 2, 3, 4, 5, 6, 7) with original detail in the initial recall, later recalled 8 critical scenes (1, 3, 4, 5, 6, 7, 8, 9) with original detail in the final recall. Six of them (1, 3, 4, 5, 6, 7) were reported as original details in both. Then, the percentage of persistent true memory for this participant is equal to 6.25% (i.e., 6 divided by 96). (2) Misinformation-induced false memory: For example, if a participant recalled 2 critical scenes (1, 3) with misinformation in the initial recall, later recalled 9 critical scenes (1, 2, 4, 5, 6, 7, 8, 9, 10) with misinformation in the final recall. Seven of these (2, 4, 5, 6, 7, 8, 9) were reported as misinformation only in the final recall. Then, the percentage of misinformation-induced false memory for this participant is equal to 7.29% (i.e., 7 divided by 96). (3) For participants who had misinformation-induced false memories in the final recall, some of them recalled the corresponding scene in the initial recall with the original detail, foil, and no-critical-detail (as underlined in the table, 0.92% + 0.15% + 2.96% = 4.02%), while others did not recall the corresponding scene in the initial recall (2.76%).
