# Supplementary material for: Shared memories of event details in the human brain are altered by misinformation and test expectations
Source: PLoS Biol. 2026 Jul 6;24(7):e3003886. doi: 10.1371/journal.pbio.3003886 (PMC13336189; doi:10.1371/journal.pbio.3003886)
Supplement: S8 Table — The underlying numerical data for this table are provided in S1 Data. (PDF) [file pbio.3003886.s011.pdf]

S8 Table. The duration in each recalled sentence for critical scenes (Mean ± SD). The underlying numerical data for this figure are provided in S1 Data.

	Initial recall	Final recall
Original	8.37 ± 1.16	7.69 ± 1.14
Misinformation	7.55 ± 2.25	8.30 ± 2.23
No-critical-detail	7.54 ± 0.92	6.66 ± 0.90
Persistent true	8.35 ± 1.27	7.71 ± 1.38
Misinformation-induced false	7.88 ± 3.02	8.29 ± 2.27
Persistent unspecific	7.77 ± 1.13	6.98 ± 1.18

Note: (1) The unit of measurement is seconds. The average duration of each sentence for each type of recall was calculated for each participant in the initial or the final recall test. (2) For the duration, there was a significant interaction between memory type (persistent true, misinformation-induced false, and persistent unspecific) and recall stage (initial and final) ($F(2, 80) = 3.82$, $p = 0.03$, $\eta^2_p = 0.09$). The main effect of memory type was also significant ($F(2, 80) = 4.13$, $p = 0.02$, $\eta^2_p = 0.09$), but the main effect of the recall stage was marginally significant ($p = 0.08$). In the initial recall, persistent true memory lasted longer than persistent unspecific memory ($p = 0.01$). In the final recall, both persistent true memory and misinformation-induced false memory lasted longer than persistent unspecific memory (*ps* < 0.01). (3) Foil recall was rare and therefore not included in the analysis.
