# Supplementary material for: Shared memories of event details in the human brain are altered by misinformation and test expectations
Source: PLoS Biol. 2026 Jul 6;24(7):e3003886. doi: 10.1371/journal.pbio.3003886 (PMC13336189; doi:10.1371/journal.pbio.3003886)
Supplement: S9 Table — The underlying numerical data for this table are provided in S1 Data. (PDF) [file pbio.3003886.s012.pdf]

S9 Table. Percentages of non-critical scenes by type of recall (%). The underlying numerical data for this figure are provided in S1 Data.

	Initial recall (Mean ± SD)	Final recall (Mean ± SD)	Initial vs. final recall *t*	*p*	Cohen's *d*
**Correct**	36.34 ± 4.90	43.45 ± 6.29	10.76	$1e^{-13}$	1.19
**Incorrect**	1.91 ± 1.27	1.64 ± 1.13	-1.81	0.08	-0.22
Unrecalled	61.75 ± 4.92	54.91 ± 6.14	-10.59	$2e^{-13}$	-1.17

Note: (1) There were a total of 304 non-critical scenes. Correct recall refers to the non-critical scene that was reported accurately. Incorrect recall refers to the non-critical scene with at least one spontaneously reported inaccurate detail. For example, if a participant recalled 38 non-critical scenes as accurate information in the initial recall, then the percentage of correct recall in the initial recall for this participant is equal to 12.5% (i.e., 38 divided by 304). (2) For non-critical scenes (i.e., post-event narratives with accurate information), correct recall was increased from the initial to the final recall, whereas incorrect recall tended to decrease. As shown in the 2 × 2 repeated measures ANOVA, there were significant main effects of recall stage (initial and final) ($F(1, 42) = 112.14$, $p = 2e^{-13}$, $\eta^2_p = 0.73$), of recall type for non-critical scenes (correct and incorrect) ($F(1, 42) = 2079.04$, $p = 2e^{-16}$, $\eta^2_p = 0.98$), as well as their interaction on the percentage ($F(1, 42) = 108.32$, $p = 3e^{-13}$, $\eta^2_p = 0.72$). (3) Confirming the misinformation effect, recall of misinformation for critical scenes increased from the initial to the final recall, whereas incorrect recall for non-critical scenes tended to decrease. As shown in the 2 × 2 repeated measures ANOVA, there were significant main effects for recall stage (initial and final) ($F(1, 42) = 68.23$, $p = 2e^{-10}$, $\eta^2_p = 0.62$), for recall type (misinformation vs. incorrect) ($F(1, 42) = 20.15$, $p = 5e^{-5}$, $\eta^2_p = 0.32$), as well as their interaction on the percentage ($F(1, 42) = 87.31$, $p = 8e^{-12}$, $\eta^2_p = 0.68$).
