# Supplementary material for: Shared memories of event details in the human brain are altered by misinformation and test expectations
Source: PLoS Biol. 2026 Jul 6;24(7):e3003886. doi: 10.1371/journal.pbio.3003886 (PMC13336189; doi:10.1371/journal.pbio.3003886)
Supplement: S10 Table — The underlying numerical data for this table are provided in S1 Data. (PDF) [file pbio.3003886.s013.pdf]

S10 Table. Inter-subject neural pattern similarity in brain regions that showed detail-specific representations between people with shared true memories (Mean ± SD). The underlying numerical data for this figure are provided in S1 Data.

Region	Same version		Different versions	
	Corresponding scene	Non-corresponding scenes	Corresponding scene	Non-corresponding scenes
During the encoding of original-event				
RAG	0.0267 ± 0.0663	0.0064 ± 0.0385	0.0161 ± 0.0436	0.0114 ± 0.0201
During the final free recall				
LVLPFC	0.0422 ± 0.0855	0.0328 ± 0.0604	0.0115 ± 0.0285	0.0117 ± 0.0205
LMTG	0.0307 ± 0.0535	0.0271 ± 0.0382	0.0222 ± 0.0449	0.0227 ± 0.0377

Note: Labels, full names, and MNI coordinates for these cortical regions: RAG: 339, right angular gyrus (x = 54, y = -52, z = 44); LVLPFC: 140, left ventrolateral prefrontal pole (x = -42, y = 48, z = -6); and LMTG: 171, left middle temporal gyrus (x = -60, y = -34, z = -4). In addition, we carried out a supplementary analysis. For each of these brain regions, we randomly selected a subset of subject pairs with shared true memories, ensuring that the number of subject pairs matched that of the number for shared false memories. We then reran the analysis. This process was repeated 10,000 times. The medians obtained from the subsample analysis were very close to the original values. This indicates that these effects are stable in both direction and magnitude. However, subsampling reduces the likelihood of achieving statistical significance. These results suggest that the design of our study, which included 43 participants and 96 critical items, was necessary to detect significant findings regarding the neural correlates of shared memories. These significant results are not likely to be obtained if future studies use a smaller sample size or include fewer critical scenes.
