# Supplementary material for: Shared memories of event details in the human brain are altered by misinformation and test expectations
Source: PLoS Biol. 2026 Jul 6;24(7):e3003886. doi: 10.1371/journal.pbio.3003886 (PMC13336189; doi:10.1371/journal.pbio.3003886)
Supplement: S11 Table — The underlying numerical data for this table are provided in S1 Data. (PDF) [file pbio.3003886.s014.pdf]

S11 Table. Inter-subject neural pattern similarity in brain regions that showed detail-specific representations between people with shared false memories (Mean ± SD). The underlying numerical data for this figure are provided in S1 Data.

Region	Same version		Different versions	
	Corresponding scene	Non-corresponding scenes	Corresponding scene	Non-corresponding scenes
During the encoding of misinformation				
RDMPFC	0.0050 ± 0.0884	-0.0065 ± 0.0329	-0.00002 ± 0.0857	0.0034 ± 0.0313
RSMG	0.0194 ± 0.0668	0.0073 ± 0.0341	-0.0010 ± 0.0569	0.0017 ± 0.0329

Note: Labels, full names, and MNI coordinates for these cortical regions: RDMPFC: 350, right dorsomedial prefrontal cortex (x = 4, y = 28, z = 48); RSMG: 286, right supramarginal gyrus (x = 60, y = -22, z = 22).
