# Supplementary material for: Shared memories of event details in the human brain are altered by misinformation and test expectations
Source: PLoS Biol. 2026 Jul 6;24(7):e3003886. doi: 10.1371/journal.pbio.3003886 (PMC13336189; doi:10.1371/journal.pbio.3003886)
Supplement: S12 Table — The underlying numerical data for this table are provided in S1 Data. (PDF) [file pbio.3003886.s015.pdf]

S12 Table. Inter-subject neural pattern similarity in the hippocampus (Mean ± SD). The underlying numerical data for this figure are provided in S1 Data.

Stage	Same version		Different versions	
	Corresponding scene	Non-corresponding scenes	Corresponding scene	Non-corresponding scenes
All participants in the recall group				
Original-event	0.0048 ± 0.0018	0.0024 ± 0.0015	0.0044 ± 0.0021	0.0022 ± 0.0016
All participants in the control group				
Original-event	0.0038 ± 0.0015	0.0016 ± 0.0009	0.0036 ± 0.0016	0.0013 ± 0.0009
Participants with shared true memories in the recall group				
Original-event	0.0055 ± 0.0481	0.0033 ± 0.0187	0.0047 ± 0.0488	0.0028 ± 0.0192
Initial recall	0.0203 ± 0.0680	0.0192 ± 0.0482	0.0193 ± 0.0658	0.0179 ± 0.0463
Misinformation	0.0038 ± 0.0479	0.0003 ± 0.0175	0.0029 ± 0.0479	0.0012 ± 0.0181
Final recall	0.0203 ± 0.0634	0.0188 ± 0.0439	0.0198 ± 0.0636	0.0180 ± 0.0442
Participants with shared false memories in the recall group				
Original-event	0.0073 ± 0.0472	0.0018 ± 0.0191	-0.0003 ± 0.0512	0.0015 ± 0.0196
Misinformation	0.0046 ± 0.0498	0.0006 ± 0.0174	-0.0004 ± 0.0504	0.0007 ± 0.0173
Final recall	0.0111 ± 0.0676	0.0100 ± 0.0416	0.0097 ± 0.0600	0.0102 ± 0.0404

Note: (1) Recall vs. Control: First, we examined the hippocampal activity patterns between participants either in the recall group or in the control group during the encoding of original events. Inter-subject similarity of hippocampal activity patterns was the dependent variable. Three independent variables were group type (i.e., recall vs. control groups), event version (i.e., same vs. different versions), and scene specificity (i.e., corresponding vs. non-corresponding scenes). A mixed-design ANOVA revealed significant main effects of scene specificity ($F(1, 98) = 673.73$, $p = 1e^{-45}$, $\eta^2_p = 0.87$), group type ($F(1, 98) = 11.35$, $p = 1e^{-3}$, $\eta^2_p = 0.10$), and event version ($F(1, 98) = 4.10$, $p = 0.046$, $\eta^2_p = 0.04$). No interaction effects reached significance (*ps* > 0.38). We observed scene-specific rather than detail-specific representations that were shared by all participants in the hippocampus during the encoding of original events. Furthermore, when participants were informed in advance that they would be taking free recall tests, the scene-specific representations they shared were significantly improved. It was in line with previous findings showing that event scenes are more easily recalled when the patterns of hippocampal activity during encoding show greater similarity across individuals [1]. Our finding supports the idea that the hippocampus combines detailed sensory information from cortical regions to form coherent representations of event scenes [2].

(2) Shared true memories: During the two encoding stages, the hippocampus displayed scene-specific representations that were shared by individuals with shared true memories who saw the same version (i.e., same version [C vs. N], $t(6068.74) = 2.74$, $p = 0.006$ and $t(6149.12) = 4.19$, $p = 3e^{-5}$, for the original-event and misinformation stages, respectively), and by those who saw different versions (i.e., different versions [C vs. N], $t(5744.67) = 2.13$, $p = 0.033$ and $t(5704.14) = 1.98$, $p = 0.047$, for the original-event and misinformation stages, respectively). However, there

was no evidence of scene-specific representations in the hippocampus shared by individuals with shared true memories during the two recall stages, regardless of whether they witnessed the same or different versions (i.e., same or different versions [C vs. N], *ps* > 0.22). Additionally, there was no evidence of detail-specific representations in the hippocampus shared by individuals with shared true memories during any of the four stages (i.e., [C minus N]: same vs. different versions, *ps* > 0.05).

(3) <u>Shared false memories</u>: During the encoding of original events, the hippocampus displayed detail-specific representations that were shared by individuals with shared false memories ($t(573) = 2.18$, $p = 0.030$, permuted $p = 0.027$). Scene-specific representations were found for viewing the same version of original events ($t(379.02) = 2.23$, $p = 0.026$), but not for the different versions ($t(279.77) = 0.63$, $p = 0.53$). During the encoding of misinformation or final recall, there no scene-specific representations (i.e., same or different versions [C vs. N]: *ps* > 0.15). Furthermore, there was no evidence of detail-specific representations shared by individuals with shared false memories in the hippocampus during these two stages (i.e., [C minus N]: same vs. different versions, *ps* > 0.19). This suggests that individuals who ultimately formed shared false memories had shared representations in their hippocampi during the encoding of original-event details.

References

1. Chen J, Leong YC, Honey CJ, Yong CH, Norman KA, Hasson U. Shared memories reveal shared structure in neural activity across individuals. Nat Neurosci. 2017;20: 115–125. doi:10.1038/nn.4450

2. Barry DN, Maguire EA. Remote memory and the hippocampus: A constructive critique. Trends Cogn Sci. 2019;23: 128–142. doi:10.1016/j.tics.2018.11.005
