# Supplementary material for: Shared memories of event details in the human brain are altered by misinformation and test expectations
Source: PLoS Biol. 2026 Jul 6;24(7):e3003886. doi: 10.1371/journal.pbio.3003886 (PMC13336189; doi:10.1371/journal.pbio.3003886)
Supplement: S1 Text — (PDF) [file pbio.3003886.s016.pdf]

S1 Text. An example of one participant's free recall of one event with scoring.
With scoring: (translated)

To facilitate understanding by English readers, we translated a participant's initial and final recall and post-event narratives with misinformation from Chinese to English. Because this was a spoken free recall task, the participant's language and logic were not perfect. The translated text retains some of the grammatical errors originally expressed by this participant. It is important to note that the scoring was based on the participants' recall in Chinese, not on the translated English text. The sentence corresponding to the event image (i.e., each scene) was labeled with a number ranging from 1 to 50 at the end. For critical details on critical scenes recalled by this participant, the original information is colored in blue and the misinformation is colored in red. (This participant viewed version A during the original-event stage).

Participant Z (Event 1: A man stole a girl's phone on the street)

**The initial free recall**
A woman wearing a black T-shirt and green pants, with a bag on each side—a white canvas tote on one side and a black leather bag on the other (scene 1). She walked toward the Xinhua Bookstore (scene 3). After purchasing several books inside the bookstore, she exited the bookstore (scene 5). Shortly after leaving the store entrance, she encountered a man wearing a grey T-shirt (scene 6). She conversed briefly with this man who wore neckband-style headphones (scene 9). She then took a book titled **Water Margin** from her white canvas bag and recommended it to him (scene 10). After chatting for a while, the woman in the black T-shirt patted the man in the grey T-shirt on the **shoulder** (scene 14). Then both of them left (scene 15). Next, a man dressed entirely in black approached from across the crosswalk (scene 18). He accidentally collided with the woman in the black T-shirt (scene 19). He was knocking her bag to the ground (scene 20). The white canvas bag fell, spilling its contents—a book, two packs of gum (one red-wrapped, one blue-wrapped)—onto the pavement (scene 20). The woman in black became furious and confronted him (scene 23). She looked extremely furious (scene 24). The man in the black T-shirt, seemed very apologetic, bent down, and both of them stooped to pick up the canvas bag from the ground (scene 26). Suddenly, the woman in the black T-shirt stood up (scene 27). She turned away, while the man in the black T-shirt took the opportunity to grab the phone from her black bag (scene 28). He swiftly gathered the items spilled from the canvas bag on the ground and handed it to the woman in black (scene 29). After the woman in black departed satisfied, he walked away (scene 33). A short while later, she realized the phone with the blue case inside her black bag was missing (scene 36). At that moment, behind the woman in black, a woman wearing a white top and skirt, carrying a dark blue backpack, stepped forward (scene 38). She spoke with the woman in black about the phone, and the woman in black suddenly understood (scene 39). She turned to look in the direction the man in the black T-shirt had left (scene 40). Finally, the woman in black and the woman in white shook hands (scene 45). Afterward, the two women left the place arm in arm (scene 47). The man in the black T-shirt hid behind a **lamp post** across the street (scene 50).

Post-event narratives (with misinformation)
A lady was walking along a sidewalk in Beijing (scene 1). The lady wore a black top and carried two bags (scene 2). Spotting a bookstore by the roadside, the lady stopped (scene 3). The lady entered the bookstore carrying a canvas bag printed with a smiley face (scene 4). The lady bought something at the bookshop and then left (scene 5). The lady stepped out of the store and ran into her male friend (scene 6). The lady waved to her male friend in greeting (scene 7). The lady's male friend happily waved back at her (scene 8). The lady chatted with her male friend for a while (scene 9). The lady showed him a book called **Dream of Red Mansion** (scene 10). The lady pointed to the bookshop she had just visited (scene 11). The lady friend expressed **confusion** about the book she bought (scene 12). The lady and her male friend chatted for a while longer (scene 13). When they said goodbye, the lady waved her **hand** to him (scene 14). The lady bid farewell to her friend and walked in the opposite direction (scene 15). As the lady passed the girl on the phone, an empty can lay at her feet (scene 16). The lady walked past a utility pole with a **coffee** flyer (scene 17). A man in black shirt crossed a street, an old lady and a little girl were nearby (scene 18). The man in black shirt collided with the lady from the **side** (scene 19). The lady's canvas bag fell to the ground, and she spread her hands (scene 20). Items spilled out of the lady's bag, including a key (scene 21). After the man and lady collided, the woman frowned and rubbed her **arm** (scene 22). The man expressed his apologies to the lady, feeling sorry (scene 23). The lady looked at the items scattered on the ground, crossing her arms in annoyance (scene 24). The man and lady crouched down to pick things up; the man wore white socks (scene 25). He placed the **red** candy box into the lady's canvas bag (scene 26). The lady stood up to check if anything else had fallen (scene 27). At that moment, the man slipped his **left** hand into her bag and stole her phone (scene 28). The man crouched on the ground, helping the lady gather the scattered items (scene 29). They stood up and brushed the dust off their clothes (scene 30). The man placed the stolen phone in his **side** pocket (scene 31). After picking up the item, the lady smiled and nodded her thanks to the man (scene 32). The lady and man parted ways, each walking in opposite directions (scene 33). The man reached the intersection, preparing to cross to the other side (scene 34). The lady continued walking when a female student approached from behind (scene 35). Feeling the sun's heat, the lady pulled a **hat** from her shoulder bag (scene 36). Suddenly, the lady realized her phone was missing (scene 37). The lady frantically searched her shoulder bag for the phone (scene 38). The female student asked what happened, and the lady said her phone was missing (scene 39). The female student pointed across the street, where a black sedan parked beside them (scene 40). The lady turned to look behind her, where a sign read “**Construction** Ahead.” (scene 41). Yet the man had vanished without a trace by then (scene 42). The lady turned back to the female student, looking utterly helpless (scene 43). With a sigh, the lady said, “The thief's long gone.” (scene 44). The lady shook hands with the female student, expressing her gratitude (scene 45). As they walked away, the student patted her shoulder in consolation (scene 46). The lady and student walked together down the street (scene 47). They turned a corner and vanished at the end of the road (scene 48). Across the street remained deserted, the traffic light still red (scene 49). After stealing the phone, the man hid behind a **tree trunk**, watching the lady (scene 50).

The final free recall of the original event
A woman wearing a black T-shirt and green pants, with a backpack on each shoulder, walked along the streets of Beijing (scene 1). Then she spotted a Xinhua Bookstore (scene 3). She entered the Xinhua Bookstore (scene 4). Soon she was out of the bookstore (scene 5). Just as she was about to leave, she ran into a male friend, who wore a grey T-shirt and black pants, sporting a pair of sports headphones (scene 6). The two chatted by the roadside (scene 9). The woman in black shirt pulled out the book **Water Margin** she had just bought from her white canvas bag (scene 10). Her male friend looked **confused** (scene 12). The woman in black shirt continued chatting with him (scene 13). When they prepared to part ways, she patted the man in grey shirt on the **shoulder** (scene 14). Then they walked off in opposite directions (scene 15). Across the street, a man wearing a black T-shirt and black pants was crossing the road (scene 18). He collided with the woman in black, accidentally knocking her canvas bag to the ground (scene 19). The book inside her bag, along with two boxes of candy—one red and one blue—all fell onto the pavement (scene 21). The woman in black shirt was furious and about to confront the man in black shirt (scene 24). Both the man and woman in black shirt immediately bent down to pick up the items from the canvas bag (scene 26). Then the woman in black shirt stood up and turned to look to the side (scene 27). At that moment, the man in black shirt secretly reached out his **left** hand and stole her phone from inside her black leather bag (scene 28). He slipped the phone into his pants' left **side** pocket (scene 31). He then picked up all the items the woman in black shirt had dropped on the ground and returned them to her (scene 29). The woman and man in black shirt parted ways, each heading in opposite directions and continuing on their paths (scene 33). After walking for a moment, the woman realized her phone was missing (scene 37). A schoolgirl wearing a white T-shirt and skirt approached from behind and pointed out to the woman that her phone seemed to have been stolen by the man in black shirt (scene 39). When the woman in black shirt turned to look, the man in black shirt had already vanished without a trace (scene 42). The woman in black shirt felt a pang of regret, but she still shook hands with the student, expressing her gratitude (scene 45). The two then walked forward arm in arm (scene 47). Meanwhile, the man in black shirt hid behind a large **tree** on the other side of the road, observing everything (scene 50).

There are two versions of the details of critical scenes in event 1 as shown below. This participant first viewed version A of the images during the original-event stage, and then read version B of the narratives during the post-event misinformation stage.

Version A	Version B	Type
Water Margin<br>(The title of a novel about heroes)	Dream of Red Mansion<br>(The title of a romance novel)	text
gestures of approval	gestures of confusion	agent
shoulder	hand	agent
flyer for tea	flyer for coffee	text
behind	sideways	location
leg	arm	agent
blue candy box	red candy box	object
right hand	left hand	agent
back	side	location
umbrella	hat	object
sign indicating school	sign indicating construction	text
behind a lamppost	behind a tree	location
